# Resilience and Protective Factors Associated with Well-Being Among Older Informal Caregivers: A Convergent Segregated Mixed Studies Systematic Review

**DOI:** 10.3390/ejihpe16090129

**Published:** 2026-08-28

**Authors:** Alba Peraza Delgado, Yurena María Rodríguez Novo, Miguel López Martínez, Mercedes Novo Muñoz

**Affiliations:** 1 Programa en Ciencias Médicas y Farmacéuticas, Desarrollo y Calidad de Vida, Universidad de La Laguna, 38200 Santa Cruz de Tenerife, Spain; alu0100994971@ull.edu.es; 2 Unidad de Investigación, Hospital Universitario Nuestra Señora de Candelaria y de la Gerencia de Atención Primaria de Tenerife, 38010 Santa Cruz de Tenerife, Spain; 3 Departamento de Enfermería, Universidad de La Laguna, 38200 Santa Cruz de Tenerife, Spain; yrodrign@ull.edu.es (Y.M.R.N.); mernov@ull.es (M.N.M.); 4 Grupo de Investigación de la ULL IENFERCAN, 38200 Santa Cruz de Tenerife, Spain; 5 Servicio de Urgencias y Emergencias, Hospital Universitario Nuestra Señora de Candelaria, 38010 Santa Cruz de Tenerife, Spain

**Keywords:** resilience, informal caregiver, social–ecological framework, well-being, older adult

## Abstract

Background: Population aging has led to an increasing proportion of older adults (aged 65 and older) acting as informal caregivers. These caregivers face risks of burden, stress, and depression. While research frequently documents negative psychological outcomes, resilience represents a crucial protective process. This systematic review synthesizes and maps the empirical evidence regarding the association between the socio-ecological resilience process and the well-being of older informal caregivers. Methods: Following Joanna Briggs Institute (JBI) mixed-methods guidelines, a convergent segregated mixed studies systematic review was conducted. A systematic search of Medline (PubMed), CINAHL, PsycINFO, and Scopus identified primary articles (2014–2025) in English and Spanish. Methodological quality was appraised using JBI critical appraisal checklists and the Mixed Methods Appraisal Tool. PROSPERO registration number: CRD420251142190. Results: Thirty-one studies were included. Elevated caregiver resilience was associated with lower reported symptoms of depression and anxiety, higher self-rated health, and positive psychological adaptation. Framed within a social ecological model, personal resources such as spirituality, hope, and self care, alongside relational assets like dyadic relationship quality and mutual coping, demonstrated a positive association with resilience and a reduction in subjective burden. Within the community context, informal peer networks and Online Health Communities functioned as essential supportive resources. On a structural level, both household wealth and the regional availability of long-term care beds acted as moderators for spousal well-being, whereas exceeding 30 weekly caregiving hours acted as a temporal threshold for positive adaptation. Conclusions: These findings suggest that resilience in older caregivers may be best conceptualized not as a static individual trait, but as a multi-level, dynamic socio-ecological process. Rather than relying on individual coping alone, public policies and clinical practice should prioritize systemic, relational, and structural environmental support, including formal respite services and long-term care infrastructure, to preserve the well-being of older spousal caregivers.

## 1. Introduction

Population aging represents one of the most significant demographic challenges of the 21st century (Alvarado García & Salazar Maya, 2014; Instituto de Mayores y Servicios Sociales, 2005). The growing global population aged 65 and over is associated with an increasing demand for long-term care. This responsibility continues to fall predominantly on informal caregivers, such as family members or friends, who constitute a fundamental pillar in supporting individuals with care dependencies (Alvira et al., 2015; Quinn & Toms, 2019). Within this demographic, older adults, defined as those aged 65 and over, represent a substantial proportion, reaching between 20% and 30% in certain countries (LaManna et al., 2020). These older caregivers, especially spouses, appear to experience a dual vulnerability: they assume intensive care tasks while simultaneously navigating the physical, emotional, and social challenges associated with their own aging process (Tkatch et al., 2017; Betini et al., 2018).

Although informal caregiving can foster positive experiences, including personal growth, deepened emotional bonds, and a sense of gratification or purpose (Bongelli et al., 2024; Quinn et al., 2019), it frequently carries a substantial psychological and physical toll (Tkatch et al., 2017). High-intensity caregiving has often been linked to elevated subjective burden, chronic sleep fragmentation, somatic burnout, social isolation, and potential declines in overall physical and mental health. These declines frequently manifest through a high prevalence of clinical anxiety and depressive symptoms (Tkatch et al., 2017; Quinn et al., 2019; Hwang & Kim, 2024; Dionne-Odom et al., 2017). The depletion of caregiver well-being does not remain localized to the individual; indeed, longitudinal research suggests that compromised caregiver health is prospectively associated with higher healthcare expenditures and increased emergency department utilization by the care recipient (Ankuda et al., 2017). This systemic risk appears to be magnified in older dyads, as their mutual and interacting age-related declines create a more fragile care environment (Bongelli et al., 2024; Ottmann & Maragoudaki, 2015; Xiong et al., 2025).

Previous research increasingly recognizes that the caregiving experience encompasses positive dimensions, such as personal growth and satisfaction (Alvira et al., 2015; J. Chen et al., 2020). Evidence suggests that the capacity to positively appraise the caregiving role tends to be associated with better mental health and fewer depressive symptoms (Quinn & Toms, 2019; Quinn et al., 2019). However, the existing literature has primarily focused on the broader dementia-caregiving population, often without isolating the specific experiences of older caregivers (aged 65 and over). Because this demographic must navigate their own age-related physical and social declines, applying general findings to them may prove insufficient. To address this gap, contemporary research has turned its attention toward protective factors and psychological adaptation in later life (Joling et al., 2016; Crespo & Fernández-Lansac, 2015).

Rather than conceptualizing resilience as a static individual trait, developmental gerontology increasingly frames resilience as a dynamic, social–ecological process of transaction between the individual and their environment (Bongelli et al., 2024; Xiong et al., 2025; H. Chen et al., 2025; Fang et al., 2022; van Roij et al., 2021). Inspired by Michael Ungar’s social–ecological model of resilience (Ungar, 2011), resilience in older caregivers is defined as the dynamic process of negotiating, managing, and adapting to significant caregiving adversity by utilizing and interacting with a range of multi-level assets and resources. Under this social–ecological paradigm, it is crucial to distinguish resilience as an interactive process from its specific operational components: contextual adversity, multi-level protective resources (antecedents), and indicators of positive adaptation (outcomes) (Joling et al., 2016; Ungar, 2011). Contextual adversity includes objective risk factors such as patient activities of daily living (ADL) limitations or neuropsychiatric symptoms. Protective resources operate nested across individual (self-efficacy, spirituality, self-care), relational (dyadic mutual coping, relationship quality), community (peer networks, online health communities), and service-system or structural levels (wealth, service access) (Joling et al., 2016; Kamalpour et al., 2021; Huenchuan & Jaspers-Faijer, 2011). Finally, positive adaptation outcomes manifest as minimized subjective burden, reduced depressive and anxiety symptoms, and preserved life satisfaction (Dionne-Odom et al., 2017; Xiong et al., 2025; H. Chen et al., 2025; Fang et al., 2022; Yan et al., 2022; Lee et al., 2022).

This social–ecological framework provides a theoretical rationale for including studies that evaluate the dynamic interaction between protective resources and adaptation outcomes, such as burden, depression, and overall well-being, under conditions of high caregiving adversity, even if those studies did not explicitly measure resilience using a standardized trait scale (Ungar, 2011). Furthermore, by structuring the narrative synthesis across the concentric levels proposed by Ungar, this review seeks to identify potentially modifiable ecological resources. Therefore, this systematic review aims to analyze the available empirical evidence regarding the association between the resilience process, its associated multi-level protective factors, and the psychological well-being of informal caregivers aged 65 and over.

## 2. Materials and Methods

This research was designed and conducted as a convergent segregated mixed-methods systematic review following the Joanna Briggs Institute (JBI) guidelines, and is reported in accordance with the Preferred Reporting Items for Systematic Reviews and Meta-Analyses (PRISMA 2020) statement (Page et al., 2021, Table S1). The review protocol was registered in the International Prospective Register of Systematic Reviews (PROSPERO; registration number: CRD420251142190). Under this framework, quantitative, qualitative, and mixed-methods evidence was extracted and synthesized in parallel and subsequently integrated to address the core research objective. This paradigm integration is theoretically grounded in our socio-ecological approach: while quantitative studies provide robust data on statistical associations and mediation pathways, qualitative evidence captures the subjective, lived experiences of older caregivers and the active strategies they employ to negotiate both formal and informal resources.

### 2.1. Eligibility Criteria

Study eligibility was defined using the PICoS (Population, Phenomenon of Interest, Context, Study Design) framework. Publication was restricted to primary, peer-reviewed articles published between 2014 and 2025 in English or Spanish. This 10-year temporal restriction is conceptually justified by the paradigm shift in gerontological resilience research around 2011–2013, which transitioned from trait-based, static personality views (e.g., hardiness) to dynamic, interactive social–ecological process models. Studies published prior to 2014 frequently utilized trait-based scales that are conceptually inconsistent with modern socio-ecological definitions. Furthermore, this timeframe aligns with international policy mandates (such as the Decade of Healthy Ageing 2021–2030 promoted by the World Health Organization [WHO]) (World Health Organization, 2020) and the recent exponential rise in clinical evaluations of digital health resources (such as Online Health Communities; Kamalpour et al., 2021) and remote psychoeducational interventions (J. Chen et al., 2020).

Eligible study populations consisted of informal, unpaid family or friend caregivers aged 65 years and older providing home-based care. For studies utilizing age-heterogeneous, mixed-age samples, inclusion was permitted only if the study: (1) exclusively evaluated older caregivers aged 65+; (2) reported disaggregated findings specifically for the older caregiver sub-cohort; or (3) reported a sample mean age of at least 65 years. In accordance with systematic methodological practices, secondary literature (e.g., systematic, scoping, or narrative reviews) was excluded from primary data extraction to prevent double-counting; such reviews were utilized for contextual background. Eligible phenomena of interest included the process of resilience, multi-level protective factors (individual, relational, community, and structural/service-system), and adaptation outcomes (reduced burden, lower depressive and anxiety symptoms, improved life satisfaction and subjective well-being) evaluated under caregiving adversity, such as patient dependency or cognitive decline.

### 2.2. Search Strategy

A comprehensive systematic search was conducted across four major electronic databases: Medline (via PubMed), Scopus, CINAHL (via EBSCO), and PsycINFO (via EBSCO), for literature published up to September 2025. To optimize sensitivity and facilitate a comprehensive retrieval, the strategy combined Medical Subject Headings (MeSH) with free-text terms, applied across title and abstract fields (e.g., [tiab] in PubMed, TITLE-ABS-KEY in Scopus, and TI or AB in CINAHL and PsycINFO). Parallel inquiries were executed in Spanish using translated terms. To minimize publication bias, a targeted exploration of grey literature was conducted between 15 and 20 September 2025, consulting OpenGrey, the WHO database, the Government of Spain’s clinical guidelines portal, and the IMSERSO (Instituto de Mayores y Servicios Sociales) database. Out of 12 grey literature records retrieved, 7 were excluded for lacking a focus on late-life resilience or populations under 65. The remaining 5 documents, including WHO briefs and national home care surveys, were selected to provide relevant contextual background for the introduction and discussion sections. The full list of query terms is detailed in Supplementary Table S1. Mendeley Reference Manager (version 2.149.0; Elsevier, Amsterdam, The Netherlands) was used for reference management and organization.

### 2.3. Screening, Selection, and Quality Appraisal

**Figure 1:**
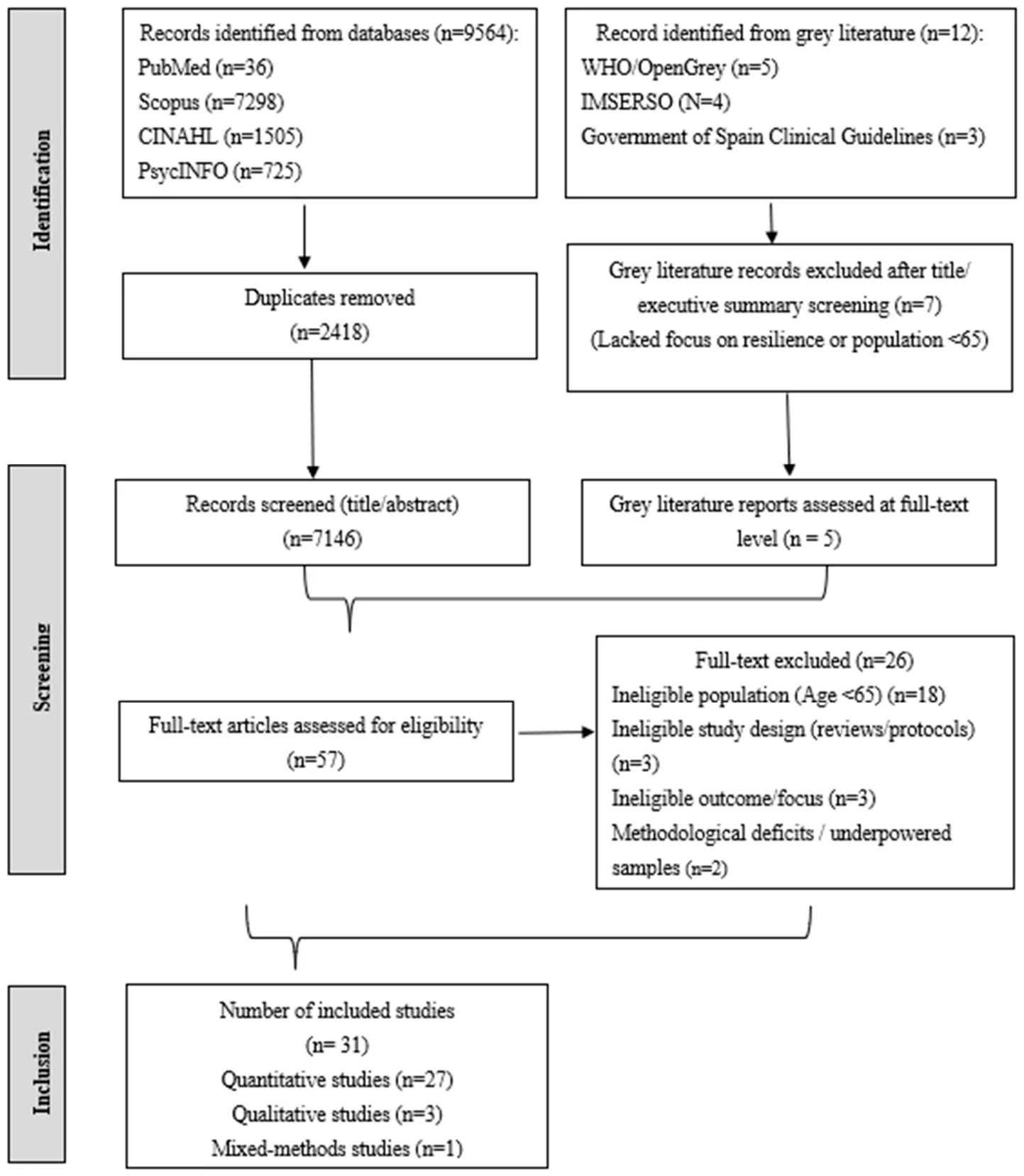
Flow chart of screening process.

The study selection process was documented transparently and is summarized in the PRISMA flow diagram (see Results, Figure 1). Two reviewers independently screened the literature by assessing titles and abstracts, followed by a full-text evaluation against the eligibility criteria. Subsequently, data were systematically collected using a pre-designed standardized form to capture essential information, including authors, publication year, methodology, caregiver demographics, measurement instruments, and main results (tabulated in the Results section, Table 1). Any discrepancies arising during these phases were resolved through discussion or, when necessary, through arbitration by a third reviewer.

Methodological quality was appraised by both reviewers using the JBI checklists and the Mixed Methods Appraisal Tool (MMAT), applying the specialized checklist tailored to each study’s design (Supplementary Table S2A,B). Specifically, analytical cross-sectional studies were evaluated for confounding factors and statistical validity; qualitative research for methodological congruency and the representation of participants’ voices; randomized controlled trials for randomization, blinding, and attrition bias; and mixed-methods studies for the rigorous integration of both components. To promote transparency, we avoided arbitrary global ratings (such as categorizing studies merely as high or moderate). Instead, appraisal decisions were documented item by item to calculate a specific compliance percentage for each study.

### 2.4. Data Synthesis

A narrative synthesis approach was primarily used due to the heterogeneity in study designs and measurement instruments. To preserve objectivity and mitigate deductive bias during synthesis, a two-stage approach was adopted. First, primary outcomes, statistical associations, and qualitative themes were inductively gathered verbatim and independently by two reviewers, without a priori reference to the theoretical framework. Second, once the primary evidence base was established, the collected data were deductively mapped onto the nested levels of Michael Ungar’s socio-ecological model. Discrepancies at both stages were resolved through discussion and the involvement of a third reviewer, thereby minimizing individual confirmation bias or the artificial shaping of primary findings to fit the conceptual structure.

## 3. Results

### 3.1. Description of the Selected Studies

The database search retrieved a total of 9564 records (PubMed: *n* = 36; Scopus: *n* = 7298; CINAHL: *n* = 1505; PsycINFO: *n* = 725), supplemented by 12 records identified through grey literature sources. After removing 2418 duplicate citations, 7146 unique records were screened based on titles and abstracts, leading to the exclusion of 7089 records that did not meet the basic eligibility criteria. During the eligibility phase, 57 full-text articles were rigorously assessed. Of these, 26 articles were excluded with specific methodological justifications (Supplementary Table S3), primarily due to ineligible population criteria (such as caregiver sample mean age under 65 years or lack of age-disaggregated data, *n* = 18), ineligible study designs (such as systematic reviews or published protocols, *n* = 3), ineligible outcomes or phenomenon of interest (such as a lack of focus on the active resilience process or subjective well-being under adversity, *n* = 3), or critical methodological deficits (such as underpowered samples or missing caregiver-specific standardized outcome measurements, *n* = 2). Consequently, 31 primary studies met all eligibility criteria and were included in the convergent segregated synthesis. Additionally, 5 grey literature reports were retained to provide structural and policy context for the narrative analysis (Figure 1).

### 3.2. Profile of the Caregivers

The descriptive analysis of the 31 included primary studies reveals a sociodemographic profile of the informal caregiver characterized by a marked female predominance, with women representing between 55.9% and 96.0% in quantitative samples (Dionne-Odom et al., 2017; Joling et al., 2016; Fang et al., 2022; Yan et al., 2022; Lee et al., 2022; Martyr et al., 2023; Gamble et al., 2022; Litzelman et al., 2016; Singh Solorzano et al., 2021; Özkan Tuncay, 2024; Freedman et al., 2019; Grosbois et al., 2022) and up to 100% in qualitative ones (White et al., 2024). The primary kinship tie predominantly involves spouses (J. Chen et al., 2020; Yan et al., 2022; Martyr et al., 2023) and, secondarily, adult children, who assume a chronic and high-intensity role (Bongelli et al., 2024; Lu et al., 2015); this demand is reflected in mean caregiving durations ranging from 1.9 to 13.4 years and time commitments frequently exceeding 30 h per week (Bongelli et al., 2024; Yan et al., 2022). Consequently, care recipients shape the context of this caregiving burden by exhibiting high levels of functional and cognitive dependency for ADL or Instrumental Activities of Daily Living (IADL), stemming primarily from diagnoses of dementia and cognitive impairment (41.9% of cases) (Joling et al., 2016; Yan et al., 2022; Martyr et al., 2023), followed by oncological (Dionne-Odom et al., 2017; J. Chen et al., 2020; Litzelman et al., 2016), cardiovascular (stroke sequelae) (Fang et al., 2022), and chronic respiratory conditions (Shah et al., 2018; Grosbois et al., 2022).

Regarding the age of the participants, the studies are divided into two main groups. The first group includes 16 studies that focused exclusively on older caregivers. These studies required participants to be at least 65 years old, or they reported a mean age above 67 years with very little variation (LaManna et al., 2020; Tkatch et al., 2017; Ankuda et al., 2017; J. Chen et al., 2020; Joling et al., 2016; H. Chen et al., 2025; Yan et al., 2022; Lee et al., 2022; Martyr et al., 2023; Gamble et al., 2022; Brandt et al., 2022; Sheehan et al., 2021; Freedman et al., 2019; Wagner & Brandt, 2018; Giebel & Sutcliffe, 2018; Van Hout et al., 2020). In these cases, caregiving occurred almost entirely between spouses who were already retired. This allowed for the study of older caregivers without the confounding influence of younger cohorts. On the other hand, a second set of 15 studies used mixed age groups (Alvira et al., 2015; Betini et al., 2018; Quinn et al., 2019; Dionne-Odom et al., 2017; Fang et al., 2022; Shah et al., 2018; White et al., 2024; Litzelman et al., 2016; Schneider & Kleindienst, 2016; Singh Solorzano et al., 2021; Lu et al., 2015; Özkan Tuncay, 2024; Li et al., 2024; Gitlin et al., 2021; Grosbois et al., 2022). Although the mean age in these works remained above 60, they permitted participants as young as 18, thereby increasing sample age variance. Consequently, these studies conflated older spousal caregivers with working adult children, who accounted for 14.7% to 62.5% of the total. This mixture makes it more difficult to accurately measure how resilience works specifically in older age.

### 3.3. Methodological Quality

The methodological quality of the studies was generally moderate to high. Out of the 31 primary studies, 13 met 100% of their respective design-specific JBI critical appraisal criteria, reflecting highly rigorous cross-sectional, qualitative, and cohort studies with robust sampling, validated measures, and controlled confounders. The remaining studies presented quality percentages ranging from 72.7% to 95.0%, with minor limitations primarily related to uncontrolled confounding factors in cross-sectional surveys, attrition rates in longitudinal cohorts, or lack of blinding in clinical trials. The quality appraisal percentages are reported in Table 1 and were systematically integrated to weight the narrative synthesis.

### 3.4. Socio-Ecological Synthesis of Late-Life Caregiving Resilience and Well-Being

**Figure 2:**
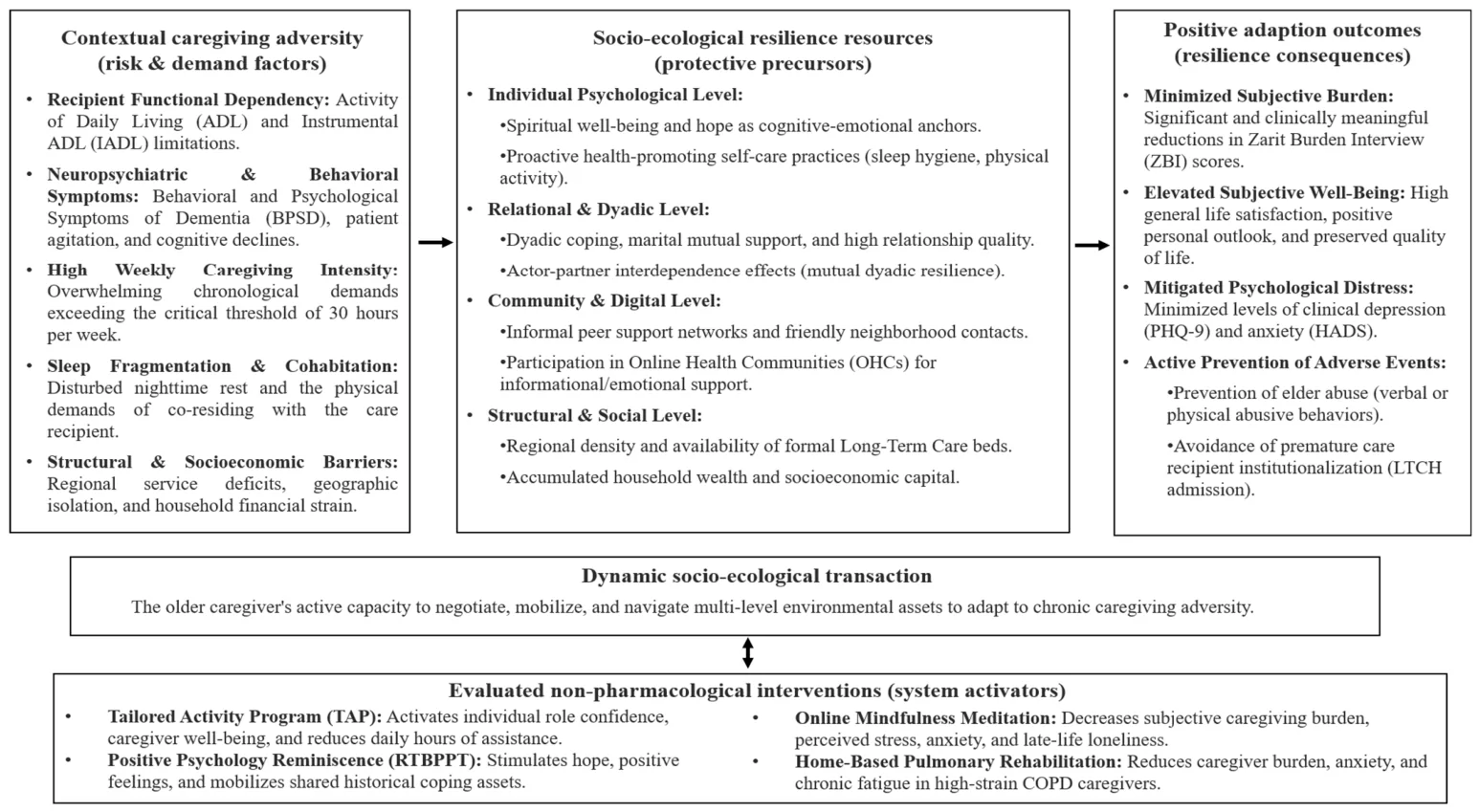
Socio-ecological categorization of empirical evidence on older caregiver resilience and well-being.

To address the conceptual limitations identified in previous studies, where resilience was frequently reduced to an isolated and static psychological trait, we synthesized the empirical findings of the 31 included studies using Michael Ungar’s socio-ecological process framework as an integrative conceptual lens. The resulting conceptual diagram (Figure 2) categorizes the late-life caregiving resilience evidence into four distinct operational components proposed by this framework, ensuring that primary findings are systematically structured without imposing unverified theoretical constructs (Ungar, 2011).

- Caregiving Adversity (Contextual Stressors): These represent the objective risk conditions that threaten caregiver well-being, driven by specific demands such as patient ADL and Instrumental Activities of Daily Living (IADL) limitations, Behavioral and Psychological Symptoms of Dementia (BPSD), cohabitation, and high-intensity caregiving hours (Schneider & Kleindienst, 2016; Lu et al., 2015; Freedman et al., 2019; Giebel & Sutcliffe, 2018; Gitlin et al., 2021).
- Socio-Ecological Resilience Resources: These represent the protective assets nested across four concentric categories: individual psychological level (self-efficacy, spirituality, hope, self-care) (Dionne-Odom et al., 2017; J. Chen et al., 2020; Özkan Tuncay, 2024); relational and dyadic level (dyadic coping, relationship quality, actor–partner support) (H. Chen et al., 2025; Martyr et al., 2023; Litzelman et al., 2016); community and digital level (peer networks, online health communities) (White et al., 2024; Li et al., 2024); and structural and service-system level (wealth, regional long-term care beds, service access) (Tkatch et al., 2017; Brandt et al., 2022; Schneider & Kleindienst, 2016; Wagner & Brandt, 2018; Gitlin et al., 2021; Grosbois et al., 2022).
- Positive Adaptation Outcomes (Resilience Outcomes): These represent the empirical indicators that successful adaptation has occurred, manifesting as minimized subjective burden as measured by the Zarit Burden Interview (ZBI), elevated subjective well-being (WHO-5, life satisfaction), and reduced depressive/anxiety symptoms (Ankuda et al., 2017; J. Chen et al., 2020; Yan et al., 2022; Lee et al., 2022; Gamble et al., 2022).
- Resilience Activators (Interventions): Non-pharmacological interventions, such as Tailored Activity Program (TAP), Positive Psychology-Based Reminiscence (RTBPPT), online mindfulness, home rehabilitation, are framed as external system facilitators that act as catalysts to strengthen existing multi-level protective factors (Tkatch et al., 2017; J. Chen et al., 2020; Gitlin et al., 2021; Grosbois et al., 2022).

### 3.5. Evidence Synthesis Across Socio-Ecological Levels

#### 3.5.1. Individual Psychological Level (Micro-System)

At the individual psychological level, the evidence suggests that internal psychological resources, self-care practices, and coping strategies act as critical protective assets against caregiving distress. Among these, spiritual well-being, sense of purpose, and caregiving preparedness consistently emerged as key predictors of lower psychological distress and improved adaptive coping across stroke and oncology caregiving contexts (Dionne-Odom et al., 2017; Özkan Tuncay, 2024).

Regarding individual resilience, primary studies indicate that a substantial proportion of caregivers sustain positive adaptation despite high adversity (J. Chen et al., 2020; Joling et al., 2016; H. Chen et al., 2025). Multivariate evidence identifies male caregiver gender, female recipient gender, higher personal mastery, and lower subjective loneliness as protective predictors of resilience, whereas cohabitation and objective care burden act as risk factors (Joling et al., 2016; Martyr et al., 2023). Furthermore, psychological resilience and active, problem-focused coping mechanisms serve as vital buffers, mitigating the direct impact of care burden on depressive symptoms and distress (Dionne-Odom et al., 2017; Fang et al., 2022; Shah et al., 2018).

However, the reviewed evidence also outlines the boundary conditions and temporal durability of these individual factors. While traits such as optimism and self-efficacy initially correlate with resilience, their independent influence on quality of life diminishes once relational quality and caregiver physical health are accounted for (Martyr et al., 2023). Finally, longitudinal evidence during macro-crises demonstrates that caregivers exhibit robust personal competence and sustained adaptive capacity over time, successfully preventing significant increases in role captivity (Gamble et al., 2022).

#### 3.5.2. Relational and Dyadic Level (Meso-System)

At the relational and dyadic level, caregiver well-being is strongly linked to the immediate dyadic environment. Across diverse populations, positive appraisals of caregiving and perceived competence consistently correlate with enhanced well-being and life satisfaction, while mitigating stress and role captivity (Alvira et al., 2015; Quinn et al., 2019; Lu et al., 2015; Van Hout et al., 2020). Conversely, relational strain and caregiver distress have direct reciprocal consequences on healthcare utilization, with spousal sadness and fatigue prospectively predicting increased emergency visits and higher healthcare expenditures for the care recipient (Ankuda et al., 2017).

Dyadic analyses highlight significant transactional effects, where social support and psychological resilience operate interdependently between caregiver and care recipient. Specifically, social support received by one member of the dyad was associated with lower depressive symptoms in both partners, with resilience potentially serving as a mutual mediating pathway (H. Chen et al., 2025). Furthermore, caregiver resilience acts as a vital buffer against relational risk, significantly lowering the likelihood of elder abuse even in high-stress scenarios characterized by severe recipient agitation and high burden (Yan et al., 2022). Social support also plays a crucial explanatory role during clinical transitions, fully mediating the link between post-surgical burden increases and caregiver depressive symptoms (Singh Solorzano et al., 2021).

Finally, primary studies indicate that relational strain is driven by behavioral demands and task unpredictability rather than mere care volume or formal diagnoses. Behavioral disturbances and the recipient’s loss of initiation in daily activities, rather than a dementia diagnosis itself, serve as the primary drivers of caregiver distress and reduced quality of life (Litzelman et al., 2016; Sheehan et al., 2021; Giebel & Sutcliffe, 2018). Similarly, task sequencing and the unpredictability of care routines, rather than total daily hours spent caregiving, represent the primary stressor impairing experienced well-being (Freedman et al., 2019).

#### 3.5.3. Community Level (Exo-System)

At the community level, older caregivers utilize informal networks and peer support to mitigate social isolation and overcome institutional gaps. Cultural and linguistic factors profoundly shape access to these community resources. For minority caregivers facing language barriers and a lack of culturally appropriate formal services, internal family solidarity and informal peer networks become the primary mechanisms of resilience (Li et al., 2024). In broader contexts, the absence of robust social and community support is consistently linked to elevated caregiver burden and depressive symptoms (White et al., 2024; Li et al., 2024). Furthermore, vulnerability to community isolation and financial strain is unevenly distributed, with female caregivers and adult offspring experiencing significantly less community support compared to their male or spousal counterparts (Lee et al., 2022; White et al., 2024; Wagner & Brandt, 2018).

#### 3.5.4. Service-System Level (Exo-System/Macro-System)

The healthcare and social services systems represent a critical exosystemic resource. The synthesized evidence demonstrates that access to structured non-pharmacological interventions is consistently associated with positive caregiver outcomes, although these benefits exhibit distinct boundary conditions. Psychosocial and behavioral interventions, such as TAP, RTBPPT and online mindfulness programs, significantly reduce caregiver burden, stress, anxiety, and loneliness while simultaneously enhancing hope and mental health-related quality of life (Tkatch et al., 2017; J. Chen et al., 2020; Gitlin et al., 2021; Grosbois et al., 2022).

However, the efficacy of these structured programs is limited by the care recipient’s clinical severity and temporal factors. For instance, extreme disease severity can restrict the well-being benefits caregivers derive from integrated home-based rehabilitation programs (Grosbois et al., 2022). Similarly, while tailored activity interventions effectively boost short-term caregiver well-being and confidence in symptom management, they may not alleviate global depressive symptoms or prevent long-term recipient agitation. This highlights that time-limited clinical programs are insufficient on their own and must be paired with continuous structural supports (Gitlin et al., 2021).

The necessity of robust, continuous service provision is further reinforced by qualitative findings. Caregiver well-being and decisions regarding residential placement are heavily dependent on their acquired knowledge and confidence in managing complex behaviors. When adequate formal service support is lacking, caregivers frequently experience severe emotional exhaustion, reaching a critical breaking point that ultimately precipitates the institutionalization of the care recipient (White et al., 2024).

#### 3.5.5. Structural and Social Level (Macrosystem)

At the macrosystemic level, caregiver well-being is heavily constrained by broader socioeconomic, regulatory, and policy factors. Socioeconomic status and educational attainment serve as fundamental structural modulators, exerting a greater overall influence on caregiver psychological well-being and depression than the caregiving role itself, establishing financial security as a prerequisite structural buffer (LaManna et al., 2020). Regional healthcare infrastructure and national policy frameworks further shape these outcomes (Wagner & Brandt, 2018; Spasova et al., 2018). Caregivers residing in regions with a higher density of long-term care facilities report significantly lower depression and loneliness, a protective effect mediated by the preservation of the caregiver’s sense of personal control (Brandt et al., 2022). Additionally, the financial strain of caregiving is deeply influenced by national models of care (Brandt et al., 2022; Schneider & Kleindienst, 2016). Caregivers in systems heavily reliant on direct out-of-pocket family expenses, such as Southern European models, experience severe financial problems directly tied to the care recipient’s degree of dependency (Alvira et al., 2015; Wagner & Brandt, 2018; Spasova et al., 2018).

Despite the protective nature of socioeconomic and regional assets, the synthesized evidence reveals a clear structural ceiling when care demands become excessive. Longitudinal data demonstrate that high household wealth is insufficient to buffer the severe decline in life satisfaction associated with providing high-intensity personal care in the home (Brandt et al., 2022). This structural limit is corroborated by temporal analyses of caregiving capacity. While caregivers can experience positive subjective well-being when providing moderate levels of assistance, exceeding a critical threshold of weekly caregiving intensity precipitates a sharp and unsustainable decline in well-being, demarcating the absolute functional limit of informal caregiving capacity (Schneider & Kleindienst, 2016).

## 4. Discussion

The findings indicate that older caregiver adaptation in late life is a complex, transaction-based process rather than a static, isolated psychological trait. Historically, caregiving research has frequently suffered from a reductionist approach, viewing resilience primarily as individual “grit” or an immutable personality profile. To address this limitation, this review adopts the socio-ecological resilience framework proposed by Michael Ungar (Ungar, 2011). Under this model, resilience is conceptualized as the capacity of individuals to navigate their social, physical, and institutional environments to negotiate for and access resources that sustain their well-being in times of adversity, combined with the capacity of their environments to provide those resources in culturally meaningful ways. This paradigm shift transitions the analytical focus from holding the individual older caregiver morally responsible for coping, to evaluating the responsiveness, accessibility, and quality of their concentric ecological systems. By mapping our empirical findings onto Ungar’s nested levels, this discussion offers a unified, systemic conceptualization of late-life caregiving resilience.

At the individual psychological level, the synthesized evidence highlights that internal cognitive–emotional resources and proactive behavioral practices are critical for initial stress moderation. Key protective assets identified across several high-quality quantitative studies include spiritual well-being, hope, and optimism, which serve as cognitive–emotional anchors that enable caregivers to reframe their experiences and find positive meaning amidst chronic strain (LaManna et al., 2020; Dionne-Odom et al., 2017; Özkan Tuncay, 2024). These cognitive assets are reinforced by the active practice of self-care. Specifically, caregivers who maintain health-promoting lifestyles, adequate sleep hygiene, physical exercise, and regular medical checkups experience significantly lower levels of somatic burnout, depression, and generalized anxiety (Dionne-Odom et al., 2017). Furthermore, positive appraisals, such as finding gratitude, purpose, and self-esteem in the caregiving role (Positive Aspects of Caregiving), act as psychological buffers that directly preserve subjective life satisfaction (Alvira et al., 2015; Quinn et al., 2019). However, in accordance with Ungar’s framework, these micro-systemic psychological traits are not infinite resources (Ungar, 2011). Individual coping strategies have a clear protective “ceiling” that is easily breached when caregiving adversity becomes structurally and chronologically overwhelming.

The meso-systemic level illustrates that resilience in late-life caregiving is inherently co-constructed within the immediate relational environment, particularly the caregiver–recipient dyad. The quality of the pre-existing relationship and the capacity for mutual dyadic coping represent major protective assets that prevent role captivity, social alienation, and caregiver depression (Martyr et al., 2023; Van Hout et al., 2020). Utilizing an advanced Actor–Partner Interdependence Model (APIM), recent evidence reveals that resilience operates through reciprocal transactional pathways, where a caregiver’s individual resilience not only buffers their own mental health but exerts a powerful partner effect that significantly reduces the depressive symptoms of the care recipient (H. Chen et al., 2025). Conversely, the relational environment also serves as a critical conduit for negative spillover. Physical exhaustion, severe sleep fragmentation, and emotional distress of spousal caregivers are prospectively and independently associated with compromised patient-perceived quality of oncology care (Litzelman et al., 2016), as well as significant increases in healthcare expenditures and emergency department utilization for the care recipient (Ankuda et al., 2017). Subjective multidimensional burden fully mediates the relationship between objective stressors, such as patient physical limitations (ADL/IADL) and demanding neuropsychiatric behaviors, and caregiver life satisfaction (Lu et al., 2015). Therefore, the older caregiver’s well-being cannot be viewed as an isolated psychological state, but must be recognized as a relational pillar that directly sustains the safety and viability of home-based care.

Older caregivers actively negotiate and draw upon external community-level assets to maintain their psychological well-being and quality of life. The cultivation of informal friendship networks, neighborhood mutual aid, and peer-to-peer caregiver support represents a vital social resource that directly alleviates the emotional isolation of caring (Lee et al., 2022; White et al., 2024). In the modern era, the community exo-system has evolved to incorporate the digital landscape. Structured participation in Online Health Communities acts as an accessible, low-barrier, community-level resource that is highly suited to older adult caregivers with limited physical mobility (Kamalpour et al., 2021). These virtual platforms foster resilience by enabling caregivers to exchange real-time emotional and informational support, validating their experiences, reducing loneliness, and promoting adaptive coping from within the home. Nevertheless, community-level resources are heavily dependent on cultural norms; in traditional or migrant communities, cultural expectations (such as filial piety) can act as an individual motivator but simultaneously prevent caregivers from seeking external formal support, exacerbating isolation (Li et al., 2024).

The formal service ecosystem represents a vital environmental resource that can proactively activate or deactivate the caregiver’s coping capacity. Non-pharmacological, evidence-based programs evaluated in the literature, such as the TAP, RTBPPT, online mindfulness training, and home-based disease management education, should be conceptualized as external systemic activators of resilience (Tkatch et al., 2017; J. Chen et al., 2020; Gitlin et al., 2021; Grosbois et al., 2022). Rather than focusing strictly on altering patient clinical parameters (e.g., modifying clinical agitation or lung capacity), the true therapeutic value of these programs lies in their capacity to restore the caregiver’s internal adaptive resources. By teaching structured, customized activity matching, or training in emotional stress-management and mindfulness, these interventions significantly enhance caregiver self-efficacy, role confidence, and subjective quality of life while notably reducing subjective burden and the daily hours of stressful physical assistance. Consequently, service-level clinical programs must be funded and implemented not as temporary patches for patients, but as strategic environmental resources designed to protect the caregiver’s psychological capital.

At the macro-systemic level, structural, economic, and policy conditions dictate the ultimate boundaries of the caregiver’s adaptive capacity. Multilevel regional analyses across Europe demonstrate that broad structural factors, specifically household wealth and regional long-term care bed density in nursing homes, directly shape caregiver life satisfaction and alleviate loneliness (Brandt et al., 2022; Wagner & Brandt, 2018). Spousal caregivers residing in regions with robust, formal care service infrastructures experience higher subjective well-being, a relationship fully mediated by their preserved sense of personal control over their lives. Furthermore, caregiving intensity, modeled through weekly care hours, represents a critical chronological ceiling: moderate commitments (under 30 h per week) can coexist with positive net utility and life satisfaction, whereas high-intensity commitments (exceeding 30 h per week) lead to an unsustainable collapse in life satisfaction (Schneider & Kleindienst, 2016). This structural threshold suggests that caregiving resilience may have functional boundaries. When environmental demands become chronologically overwhelming and socioeconomic or policy support is lacking, individual and dyadic psychological coping strategies may be insufficient, indicating that macro-level infrastructure can be a crucial determinant of late-life caregiver adaptation.

A critical methodological debate emerging from this systematic review concerns the age composition of the included informal caregiver samples. In late-life caregiving research, studies frequently employ a sample mean age above 65 years as a proxy for “older caregivers” without establishing age-homogeneity. To ensure empirical precision, this discussion establishes a clear distinction between: (1) studies focusing on strictly older caregiver samples (aged ≥ 65 years) (LaManna et al., 2020; Ankuda et al., 2017; Sheehan et al., 2021), which capture true late-life developmental contexts; and (2) studies examining highly age-heterogeneous, mixed samples (Dionne-Odom et al., 2017; Fang et al., 2022; Özkan Tuncay, 2024; Grosbois et al., 2022), which met the inclusion criteria via a sample mean age above 65 years but included substantial portions of younger adult children. Applying findings from age-heterogeneous samples to older caregivers risks concealing unique age-specific adaptation mechanisms.

The neurobiological and clinical significance of this age aggregation bias is powerfully illustrated by the work of Smagula et al. (Smagula et al., 2017), who examined how brain structural markers and caregiving characteristics interact as correlates of caregiving strain. Older caregivers, owing to normal and pathological biological aging, exhibit a higher prevalence of subclinical brain structural changes, such as white matter hyperintensities and cerebral atrophy, which can interact with the chronic, daily stressors of caregiving to accelerate cognitive and emotional depletion. When research aggregates younger and older caregivers into a single sample, these critical, age-dependent neurobiological and physiological mechanisms are completely concealed. Such aggregated analyses fail to capture how the intersection of neurological aging and chronic caregiver distress increases vulnerability to clinical depression and burden specifically in caregivers over 65. Citing Smagula et al. therefore provides a compelling, objective justification for why age-aggregated literature is limited: it masks the specific neurobiological and neural vulnerabilities that uniquely characterize older caregiving cohorts, underscoring the urgent necessity of utilizing age-homogeneous samples in caregiver resilience research (Smagula et al., 2017).

The developmental, physical, and socioeconomic realities of spousal caregivers (typically aged ≥ 65 years) differ fundamentally from those of younger adult children (typically aged < 60 years). Younger caregivers face intense socioeconomic role conflicts, specifically balancing high caregiving demands with active employment, financial accumulation, and raising dependent children (Lee et al., 2022). In contrast, older caregivers (especially spousal caregivers) face age-related physical and cognitive declines, higher baseline vulnerability to chronic conditions, sleep fragmentation, and the profound emotional distress of spousal bereavement (Freedman et al., 2019; Van Hout et al., 2020). While the inclusion of mixed-age samples with mean ages above 65 years was necessary to ensure a comprehensive scope of the literature, this heterogeneity represents a major limitation that must be recognized. Attributing resilience outcomes uniformly to all caregivers obscures the specific physiological, social, and policy requirements of true older adult caregivers. Consequently, future research must systematically prioritize age-disaggregated analyses, explicitly reporting separate baseline and outcome scores for caregivers aged < 65, 65–79, and 80+.

A key methodological consideration of this review is the inclusion of studies that did not utilize standardized individual resilience scales (e.g., CD-RISC). While this decision introduces methodological heterogeneity, it is conceptually justified by our socio-ecological paradigm. From a transactional perspective, resilience is not a static psychological trait confined to individual ‘grit’; rather, it is a dynamic process of negotiating and accessing multi-level resources under adversity. Restricting our inclusion criteria to studies employing individual trait-based resilience scales would have introduced an individualistic selection bias, overlooking crucial qualitative and quantitative evidence on relational, community, and structural protective factors that actively sustain caregiver well-being. Nevertheless, we acknowledge that synthesizing studies with diverse operationalizations of resilience (trait vs. process/resource-based) limits direct statistical comparison and requires cautious interpretation of the synthesized findings.

### Recommendations for Research, Clinical Practice, and Public Policy

To translate this socio-ecological synthesis into actionable progress, we propose three core multi-level recommendations for researchers, clinicians, and policy makers:

- Systemic Policy Shift from Individual Grit to Environmental Support: Public policies must transition from merely offering individual psychological counseling or assuming family availability, to actively building supportive, responsive environments. This shift requires formal investment in community-based respite infrastructures, adult day centers, and culturally-adapted home care training, as well as expanding regional long-term care bed availability to preserve older caregivers’ sense of personal control and subjective life satisfaction.
- Clinical Adoption of Dyadic-Centered Interventions: Clinicians must recognize the dyadic interdependence of late-life care. Interventions should be co-designed to target the care recipient and the primary caregiver as a singular, interactive unit. Programs must actively focus on strengthening relational protective assets, such as dyadic coping, mutual relationship quality, and joint communication, rather than training caregivers in isolation. Given the significant actor–partner effects, enhancing the resilience of one partner directly buffers the psychological distress of the other, preserving relational safety.
- Mandatory Methodological Disaggregation of Age Data: To eliminate the age composition bias in future research, funding bodies, journal editors, and trial registries should mandate the disaggregation of demographic and outcome scores specifically for older age cohorts. Researchers must report separate baselines, standard deviations, and treatment outcomes for distinct developmental groups (e.g., <65, 65–79, and ≥80 years). This methodological precision is essential to design and deploy interventions tailored specifically to the unique physiological, social, and chronological realities of older adult spousal caregivers.

## 5. Conclusions

The present study demonstrates that resilience is a significant protective factor in informal caregivers aged 65 and over, being consistently associated with better psychological outcomes, reduced subjective burden, and greater overall well-being. Multi-level resources, including individual self-efficacy, spiritual well-being, hope, and proactive self-care practices, were linked to less depression and anxiety, while relational assets (such as dyadic coping and relationship quality) promote positive, mutual adaptation within the caregiving dyad.

Caregiver burden was strongly associated with compromised well-being and depressive symptoms, with the severity of this burden being linked to contextual risk factors such as low household income, high weekly care hours, and continuous cohabitation with the care recipient. Although older spousal caregivers are more vulnerable to age-related functional and physical declines, their accumulated life experience and spiritual coping were associated with a partial mitigation of these difficulties. It is important to emphasize that resilience is not an isolated individual trait, but rather the result of a complex, dynamic transaction between the caregiver’s personal resources and their external environment, particularly informal social support, digital peer networks, and formal respite service-system access.

Consequently, future clinical practice and research must transition from holding the individual older caregiver morally responsible for coping, and focus on designing and deploying effective dyadic and systemic interventions. Public policies are urgently needed to guarantee equitable access to formal environmental support, such as community respite infrastructure, regional long-term care bed availability, and age friendly neighborhoods, which represents an indispensable factor in sustaining the well-being of older caregivers and the safety of home-based care systems.

## Figures and Tables

**Table 1:** Synthesis of Included Primary Studies.

Author, Year & Ref.	Country & Study Design	Sample Characteristics (*n*)	Age	Key Measures & Instruments	Main Findings & Outcomes	JBI Score (%)
Block A: Studies Directly Measuring Resilience						
**(Martyr et al., 2023)**	United Kingdom. Cross-sectional study.	*n* = 1222 informal caregivers of people with mild-to-moderate dementia (81.9% spouses/partners, 69.7% female).	Mean caregiver age: 69.02 ± 10.98 years.	Composite Resilience Score derived from 5 instruments (Relative Stress Scale [RSS], Role Captivity Scale, Positive Aspects of Caregiving [PAC], NPI-Q Caregiver Distress, and a Stressful Life Events Schedule). Predictors modeled: Lubben Social Network Scale, Mini-IPIP personality, caregiver competence scale, Pfeffer FAQ, Generalized Self-Efficacy Scale, Life Orientation Test-Revised, Rosenberg Self-Esteem Scale.	Caregivers demonstrated high overall resilience. In the multivariable model, greater resilience was significantly predicted by sociocultural and contextual factors (being older, male, caring for an older recipient with fewer functional and neuropsychiatric symptoms, better dyadic relationship quality, and fewer social restrictions), along with psychological factors (lower neuroticism and higher caregiver competence). Self-efficacy, optimism, and self-esteem were not significant predictors.	100.0%
**(H. Chen et al., 2025)**	China. Cross-sectional study.	*n* = 425 dyads (850 participants).	Caregivers’ mean age: 68.95 ± 12.28 years.	14-Item Resilience Scale, Multidimensional Scale of Perceived Social Support, Patient Health Questionnaire-9 (depression).	Perceived social support reduces depression via resilience for both caregivers and older adults. Crucially, an asymmetrical partner effect revealed that caregiver resilience significantly mitigates the older adult’s depression, but the reverse is not true. This highlights the caregiver as the vital emotional anchor of the dyad.	100.0%
**(Yan et al., 2022)**	Hong Kong. Cross-sectional study.	*n* = 600 family caregivers of community-dwelling older adults (77.9% spouses, 19.3% children, 67.4% female).	Mean caregiver age: 71.04 ± 10.59 years.	Connor-Davidson Resilience Scale (CD-RISC-10), Zarit Burden Interview (ZBI-22), Cohen-Mansfield Agitation Inventory, Generalised Self-Efficacy Scale, Wills Social Support Scale, Revised Conflict Tactics Scale, Older Adults Financial Exploitation Measure.	Higher caregiver resilience acts as a vital protective asset against elder abuse. Resilience directly mitigates the impact of care recipient behavioral symptoms and subjective burden on abusive verbal or physical behaviors.	100.0%
**(Fang et al., 2022)**	China. Cross-sectional study.	*n* = 245 family caregivers of stroke survivors (75.1% spouses, 78.78% female).	Mean caregiver age: 59.05 years. Age distribution: <55 (33.88%, *n* = 83); 55–64 (31.43%, *n* = 77); 65–74 (28.98%, *n* = 71); and ≥75 (5.71%, *n* = 14).	Connor-Davidson Resilience Scale (CD-RISC-25), Zarit Caregiver Burden Interview (ZBI-22), Center for Epidemiologic Studies Depression Scale (CES-D), Barthel Index (BI).	A high prevalence of depression (72.65%) was observed among caregivers. While care burden directly increases depressive symptoms, resilience acts as a significant protective buffer. It partially mediates this relationship, mitigating 26.32% of the negative impact that care burden has on depression.	100.0%
**(Joling et al., 2016)**	Netherlands & United Kingdom. Cross-sectional study.	*n* = 1048 primary informal caregivers (59.4% spouses, 69.6% female)	Mean caregiver age: 66.3 ± 12.5 years.	General Health Questionnaire (GHQ-12/28 for well-being). Adversities: MMSE/CDR/GDS (dementia severity), EQ-5D/ASEP (self-care), CSDD/NPI-M (mood/behavioral problems), weekly hours spent caring (substantial care role ≥ 40 h).	Resilience prevalence ranged from 35.2% to 43.4%. Higher resilience was significantly predicted by being a male caregiver, caring for a female patient, having higher mastery and competence, and experiencing less loneliness. Conversely, higher care burden and cohabiting with the patient reduced resilience, while coping styles and case management had no significant effect.	100.0%
**(Shah et al., 2018)**	United Kingdom. Mixed-methods study.	*n* = 37 unique informal caregivers (81% spouses, 65% female).	Mean caregiver age: 66 ± 13 years.	CD-RISC-10 (Resilience), Zarit Burden Interview (ZBI-22), Short-Form Health Survey (SF-36) version 1.	Caregivers of interstitial lung disease (ILD) patients face high burden (mean ZBI = 21.0). Higher resilience and active, problem-focused coping strategies (planning, active support seeking) significantly buffer caregiving distress.	95.0% (MMAT:5/5)
Block B: Studies evaluating related protective factors and caregiver well-being						
**(White et al., 2024)**	Australia. Qualitative study.	*n* = 25	Caregiver age range: 43–82 years (11 are ≥65 years, 14 are <65).	Semi-structured interviews, thematic inductive analysis.	Three core themes emerged: severe caregiver exhaustion and isolation; complex, guilt-inducing care transition decisions (followed by relief); and challenging post-transition adjustments involving grief, especially for spouses. The findings highlight critical gaps in post-diagnostic education, navigation support, and early care planning.	100.0%
**(Gamble et al., 2022)**	United Kingdom. Cohort study.	*n* = 468 family caregivers. Comprises a matched pre-pandemic cohort (*n* = 312) and a pandemic cohort (*n* = 156). 89.7% spouses/partners, 62.8–63.1% female.	Mean caregiver age: ~69.0 years. Age distribution: <65 (23.4–28.2%), 65–69 (25.0%), 70–74 (18.0–23.4%), 75–79 (12.8–13.5%), and ≥80 years (14.7–16.0%).	Single item for Well-being (WHO-5 spirits item), single item for Quality of Life (WHOQOL-BREF item), single item for Coping (COPE Index item), Zarit-derived Competence Scale (3 items, score 0–12), Pearlin Role Captivity Scale (3 items, score 0–12).	While well-being and QoL declined similarly across both cohorts over time, the pandemic cohort demonstrated significantly superior coping, more stable competency, and a smaller increase in role captivity. These findings highlight strong long-term resilience and active adaptation during a prolonged societal crisis.	77.27%
**(Ankuda et al., 2017)**	United States. Prospective Cohort study.	*n* = 3101 spousal caregiver–recipient dyads (5960 longitudinal observations).	Mean caregiver age: 74.17 ± 0.24 years.	CES-D-8 (Depression), Jenkins Sleep Scale, single-item General Self-Rated Health, and single-item Severe Fatigue measure.	Worse caregiver well-being, characterized by clinically significant fatigue and sadness, is prospectively and independently associated with a decline in caregiving capacity and dyadic stability, leading to an increased risk of crisis-driven healthcare transitions (such as emergency department visits).	90.9%
**(Brandt et al., 2022)**	Europe (17 countries). Cohort study (SHARE, waves 2, 4, 5, and 6).	*n* = 154,306 person-year observations from 79,014 individuals (including. *n* = 12,526 within-household caregivers) aged ≥ 50 across 17 European nations.	Mean caregiver age: 67.48 ± 10.05 years.	Single-item Life Satisfaction scale (0–10), regular co-resident personal care provision (personal care regularly last 12 months), and Household Wealth quintiles (sum of net financial and real assets).	In-home caregiving significantly reduced life satisfaction. While household wealth was protective for baseline satisfaction, it did not moderate the negative impact of caregiving. This demonstrates that the burden of caregiving is universally detrimental to life satisfaction, regardless of socioeconomic status.	81.81%
**(Betini et al., 2018)**	Canada. Cohort/Instrument Validation	Derivation (*n* = 362): Informal caregivers | Community clients (MAPLe 4–5).	Derivation caregivers’ mean age: 64.1 ± 13.1 years. Validation caregivers: 5% <45, 34% 45–64, 21% 65–74, and 39% ≥75 years.	Caregiver Wellbeing Index (CWBI) (4 self-reported items on 3-day frequency of: anhedonia, anxiety, sadness, and feeling overwhelmed; score 0–8). interRAI Home Care and interRAI Community Health Assessment.	Poor caregiver wellbeing (reliably measured by the CWBI) is strongly associated with distress, family conflict, and patient behavioral symptoms. Crucially, it significantly predicts the patient’s future admission to a long-term care facility (OR = 3.52).	86.36%
		Validation (*n* = 1020): Primary caregivers|Home care clients (85% co-residing).				
**(Litzelman et al., 2016)**	United States. Prospective Cohort study.	*n* = 689 dyads (62.15% spouses, 78.99% female).	Caregivers’ age distribution: 20–50 (26.91%), 51–60 (28.40%), 61–70 (24.36%), and ≥71 years (20.33%).	10-item Center for Epidemiologic Studies Depression Scale (CESD-10) and single-item Self-Rated Health.	Caregiver depression and poor self-rated health significantly predict patient-reported poor Quality of Care. This risk increases nearly five-fold when the caregiver experiences both. Physician communication and care coordination appear to mediate this negative relationship.	81.81%
**(Schneider & Kleindienst, 2016)**	Europe (12 countries). Cross-sectional survey (SHARE Wave 2).	*n* = 29,471.	Mean caregiver age: 66.8 years.	Subjective Life Satisfaction scale (0–10), Log household income, weekly care hours.	Moderate caregiving (10–30 h/week) and personal care tasks increase life satisfaction. Conversely, high-intensity care (>30 h/week) and caring for individuals of the same or a younger generation decrease life satisfaction and are more emotionally taxing.	87.5%
**(Singh Solorzano et al., 2021)**	United Kingdom. Prospective Cohort study.	*n* = 101 (96.0% female, predominantly spouses/partners)	Mean caregiver age: 65.89 ± 8.48 years (range: 39–88).	Oberst Caregiver Burden Scale, ENRICHD Social Support Instrument (ESSI, 6-item version), Beck Depression Inventory, CASP-19 (Control, Autonomy, Self-Realisation, Pleasure scale).	Post-surgery, caregiver burden increased and social support decreased. Crucially, this decline in social support fully mediated the negative impact of increased care burden on the caregiver’s depression and subjective well-being.	72.7%
**(Lee et al., 2022)**	Australia. Cross-sectional Study.	*n* = 189 (83.5% female, 56.2% spouses).	Mean caregiver age: 68 ± 9.3 years (range: 50–90+).	Dementia Quality of Life Scale for Older Family Carers (22 items, score 22–110), 5-point Likert self-rated health, 11-point Visual Analogue Scale (0–10) for health importance, and closed-ended barrier checklists.	Older carers actively employ diverse physical, mental, and social strategies (such as walking and peer support networks) to maintain QoL. Cohabitation and caring for a patient with depression/anxiety are major risk factors.	100.0%
**(Dionne-Odom et al., 2017)**	United States. Cross-sectional Study.	*n* = 294 (72.8% female, 60.2% spouses/partners).	Mean caregiver age: 65.5 ± 12.7 years (range: 20–90).	Health Promoting Lifestyle Profile II (52 items), Hospital Anxiety and Depression Scale (HADS), Short Form 12 Health Survey (SF-12v2), Preparedness for Caregiving Scale, Family Decision-Making Self-Efficacy Scale.	High rates of caregiver depression (23%) and anxiety (34%) were observed. Poor self-care directly worsens caregiver mental health and Quality of Life. Crucially, caregiver self-neglect reduces their care preparedness and decision-making confidence, undermining their ability to manage patient needs.	87.5%
**(LaManna et al., 2020)**	United States. Cross-sectional study.	*n* = 186 community-dwelling older adults (62.37% female): *n* = 65 current caregivers, *n* = 52 former caregivers (aged ≥ 65 during caregiving), and *n* = 69 never caregivers.	Mean age: 69.93 ± 4.57 years (current: 68.52 ± 5.90; former: 71.81 ± 5.56; never: 69.93 ± 4.57).	10-item Center for Epidemiological Studies Depression Scale (CESD-10), single-item Health Satisfaction scale (0–4), 6-item Rapid Caregiver Well-Being Scale (R-CWBS; range 0–4), and a 15-item Health Problems Index.	Current caregivers report significantly compromised self-rated health and well-being compared to former or never-caregivers. Household income has a much stronger overall influence on well-being than caregiving status itself.	93.75%
**(Lu et al., 2015)**	China. Cross-sectional study.	*n* = 494	Mean caregiver age: 62.65 ± 11.89 years. Caregivers’ age distribution: <60 years: 50.6% (*n* = 250) 60–74 years: 27.1% (*n* = 134) 75–84 years: 16.8% (*n* = 83) ≥85 years: 4.7% (*n* = 23).	24-item Chinese Caregiver Burden Inventory (CBI; time-dependence, physical, social, emotional, and developmental subscales), Barthel Index (BI), Short Portable Mental Status Questionnaire, 5-item elder behavioral problems scale, 20-item CES-D, and 5-item Satisfaction with Life Scale (SWLS).	Objective stressors (recipient’s ADL limits and behavioral problems) are associated with multidimensional caregiver burden. Subjective burden (physical, social, and developmental dimensions) fully mediates the relationship between objective stressors and caregivers’ subjective well-being (depressive symptoms and life satisfaction). Behavioral problems represent the most demanding stressor, affecting all five burden dimensions.	100.0%
**(Özkan Tuncay, 2024)**	Türkiye. Cross-sectional study.	*n* = 130 (70.0% female, 33.1% spouses).	Mean caregiver age: 47.95 ± 12.41 years (age distribution: 31.5% aged 22–44, 34.6% aged 45–64, and 33.9% aged ≥ 65)	Preparedness for Caregiving Scale (PCS; 8 items, score 0–32), 29-item Spiritual Well-Being Scale and Modified Barthel Index (score 0–100).	Spiritual well-being directly predicts preparedness (B = 0.144, *p* < 0.001). Crucially, age-group comparisons showed that older caregivers (≥65 years) report high spiritual well-being (114.77 ± 14.86) and moderate preparedness (20.75 ± 7.02) statistically equivalent to younger cohorts (*p* = 0.287 and *p* = 0.661, respectively), supporting active coping in late-life.	100.0%
**(Sheehan et al., 2021)**	United States. Cross-sectional study.	*n* = 251 (117 caring for dementia patients; 134 for non-dementia).	Caregivers’ mean age: 73.1 ± 8.3 years (dementia) vs. 70.7 ± 7.7 (non-dementia).	AD8 dementia screener, Katz ADL, Lawton IADL, 24-item Revised Memory and Behavior Problems Checklist, Schulz & Beach 1-item caregiving strain, 3-item NSOC difficulty scales, PSS-4, 4-item Treatment Burden scale, 10-item CES-D, and SF-12 (MCS/PCS).	Dementia caregivers report more care hours and initial strain. However, multivariable models show that caregiver distress is driven entirely by the patient’s total physical and behavioral problem load, rather than the specific diagnosis of dementia.	100.0%
**(Freedman et al., 2019)**	United States. Cross-sectional study.	*n* = 511 time diaries from 351 informal caregivers (55.9% female, 58.8% spousal)	Mean caregiver age: 69.5 years.	24-h retrospective time diaries (15-min intervals measuring household, personal care, visiting, and transport). Experienced Well-being scale (averaging 5 momentary emotions: calm, happy, sad, frustrated, worried across randomized activities; alpha = 0.86).	The specific daily pattern and sequence of caregiving tasks significantly impacts well-being, whereas the total amount of daily care hours does not. Specifically, being a sole caregiver or having a “marginal” care routine predicts lower experienced well-being.	100.0%
**(Quinn et al., 2019)**	United Kingdom. Cross-sectional study.	*n* = 1283 (68.7% female, 81.0% spousal).	Caregivers’ age distribution: <65 (28.8%), 65–69 (16.2%), 70–74 (20.8%), 75–79 (17.4%), and ≥80 years (16.8%).	3-item Caregiving Competence scale (score 3–12, alpha = 0.88), 9-item Positive Aspects of Caregiving (PAC) scale (score 9–45, alpha = 0.91), 15-item Relative Stress Scale (RSS; score 0–60, alpha = 0.89), 3-item Role Captivity scale (score 3–12, alpha = 0.84). Outcomes: WHO-5 Well-Being Index (transformed to 0–100, alpha = 0.86) and Satisfaction with Life Scale (SwLS; score 5–35, alpha = 0.88).	Positive caregiving dimensions (finding meaning) and competence boost life satisfaction and well-being. Crucially, these positive resources buffer the negative effects of stress and role captivity, making them key targets for resilience interventions.	100.0%
**(Wagner & Brandt, 2018)**	Europe (11 countries). Cross-sectional study.	*n* = 29,458 individuals total (including 1807 spousal caregivers).	Mean caregiver age: 69.50 ± 9.9 years.	Regional proxy for formal care: institutionalized long-term care beds per 100 people aged ≥ 65 in 2012. Outcomes: single-item life satisfaction (0–10), 3-item UCLA loneliness scale, and 16-item EURO-D depression scale. Mediator: 3-item CASP control subscale (range 3–12).	Spousal caregivers experience worse mental health and lower life satisfaction. However, regional availability of long-term care beds buffers these negative effects. The key mechanism is that having formal care alternatives enhances the caregiver’s “perceived control” over their life, which directly protects their well-being.	93.75%
**(Giebel & Sutcliffe, 2018)**	United Kingdom. Cross-sectional study.	*n* = 272 (66.5% female, 67.3% spouses).	Mean caregiver age: 67 ± 12 years.	Revised Interview for Deteriorations in Daily Living Activities in Dementia 2; separating 17 initiative and 20 performance items), Adult Carer Quality of Life, General Health Questionnaire 12 (GHQ-12; max 36), and Quality of Life in Alzheimer’s Disease.	Carer well-being is strongly associated with the patient’s capacity to initiate activities of daily living (ADL), rather than just the physical performance. Supporting patient initiative reduces caregiver stress.	75.0%
**(Alvira et al., 2015)**	Europe (8 countries). Cross-sectional study.	*n* = 2014.	Caregiver mean age ranged from 56.8 ± 13.4 years (Estonia) to 66.6 ± 13.5 years (Spain).	Caregiver Reaction Assessment; self-esteem, lack of family support, financial problems, disrupted schedule, health problems), Zarit Burden Interview (ZBI), EuroQol 5-Dimension (EQ-5D), General Health Questionnaire (GHQ-12), Neuropsychiatric Inventory Questionnaire (NPI-Q), Charlson Comorbidity Index, and Katz Index of ADL.	The negative impacts of caregiving are stronger in Home Care (HC) than in Long-Term Care Institutions. Across all countries, caregiver health issues and disrupted schedules strongly worsen burden, Quality of Life, and well-being, whereas self-esteem acts as a universal protective factor in HC. In Spain specifically, financial problems are strongly linked to patient dependency and comorbidity, reflecting high out-of-pocket costs for families.	81.25%
**(Li et al., 2024)**	New Zealand. Qualitative Study.	*n* = 12 female Chinese family caregivers (66.7% spouses).	Caregivers’ age distribution: <65 years (33.3%, *n* = 4) and ≥65 years (66.7%, *n* = 8).	Semi-structured telephone interviews, Resilience Resources Framework.	Four key themes emerged: social isolation, emotional loneliness, caregiving ambivalence (high stress buffered by family duty), and unmet needs (suspended respite services, language barriers, and lack of culturally appropriate care). Ultimately, the absence of community support severely threatens the sustainability of informal care.	100.0%
**(Van Hout et al., 2020)**	Belgium. Qualitative Study.	*n* = 11 recently bereaved former spousal caregivers (63.64% female).	Mean caregiver age: 76 years (range: 67–85 years).	Interviews. The use of standardized scales or measurement tools is not specified.	Caregivers oscillate between loss- and restoration-oriented coping, experiencing both relief and profound loneliness. This loneliness is heavily compounded by the abrupt withdrawal of healthcare professionals, who previously formed their main social network. While mastering new tasks builds self-efficacy, caregivers often suppress their grief to avoid burdening their children.	100.0%
BLOCK C: Intervention studies targeting caregiver resilience & well-being						
**(Gitlin et al., 2021)**	United States. Randomized Controlled Trial (RCT).	*n* = 250 dyads (*n* = 124 intervention, *n* = 126 control); (81.2% female, 45.6% spouses).	Mean caregiver age: 65.37 ± 12.64 years	Patient agitation and aggression (NPI-C), patient functional dependence, caregiver well-being, confidence and tracking of 6-month severe health events (death, hospitalization, depression/suicidal ideation).	At 3 months, the Tailored Activity Program (TAP) significantly improved caregiver well-being and confidence in managing symptoms. The intervention acts as an external resilience activator, reducing daily hands-on care hours and preserving the caregiver’s psychological resources.	73.07%
**(J. Chen et al., 2020)**	China. RCT.	*n* = 56 spousal caregivers of advanced cancer patients (Intervention: *n* = 27; Control: *n* = 29).	Mean caregiver age: 69.15 ± 6.60 years (experimental group) vs. 66.38 ± 5.09 years (control group).	Zarit Burden Interview (ZBI-22), Positive Aspects of Caregiving (PAC), Herth Hope Index (HHI).	An 8-session, 1-on-1 Reminiscence Therapy Based on Positive Psychology Theory (RTBPPT) significantly reduced caregiver burden (mean ZBI from 39.22 to 29.0, *p* < 0.01). It also significantly enhanced spousal caregivers’ positive feelings toward caregiving (mean PAC from 29.56 to 36.04, *p* < 0.01) and levels of hope (mean HHI from 32.96 to 40.67, *p* < 0.01).	73.07%
**(Tkatch et al., 2017)**	United States. Quasi-experimental Study (Pre-Post).	*n* = 40 enrolled older caregivers (*n* = 22 completed both pre and post surveys).	Baseline sample mean age: 71.0 years. Matched cohort mean age: 73.0 years.	Zarit Short Burden Interview (12-item ZSBI), VR-12 Health Survey (Mental/Physical Component Scores), Perceived Stress Scale (PSS-10), UCLA 3-item Loneliness Scale, GAD-7 (Anxiety).	Online mindfulness can be an effective tool for improving the psychological well-being of caregivers. It reduced caregiver burden, stress, anxiety, and loneliness, improved mental and social well-being, and proved to be feasible with high participation and retention.	77.78%
**(Grosbois et al., 2022)**	France. Quasi-experimental Study (Pre-Post).	*n* = 138 (70.3% female, 87.7% spousal).	Mean caregiver age: 60.3 ± 14.8 years. Includes disaggregated multivariate sub-cohort analysis for caregivers aged > 70 years.	Zarit Burden Interview (ZBI-22), Hospital Anxiety and Depression Scale (HADS), Fatigue Assessment Scale.	Post-intervention, caregivers showed significant reductions in burden, anxiety, depression, and fatigue. Crucially, caregiver participation led to greater improvements in patient anxiety and fatigue. The intervention was most effective at reducing burden for caregivers of patients with severe clinical conditions.	83.3%

Table 1 is physically structured to be read from left to right. All values and outcomes correspond strictly to informal caregiver samples. Quality of primary studies was appraised using JBI tools, except for the mixed-methods design, which was evaluated using the Mixed Methods Appraisal Tool (MMAT). For this study, the MMAT Category 5 (mixed-methods integration) score is provided in parentheses alongside the overall descriptive quality score. Abbreviations: JBI: Joanna Briggs Institute; N: Sample size; RSS: Relative Stress Scale; PAC: Positive Aspects of Caregiving; NPI-Q/NPI-C/NPI-M: Neuropsychiatric Inventory (Questionnaire/Clinician/Mood); Mini-IPIP: Mini-International Personality Item Pool; FAQ: Pfeffer Functional Activities Questionnaire; CD-RISC (includes 10 and 25 versions): Connor-Davidson Resilience Scale; ZBI/ZBI-22: Zarit Burden Interview; CES-D (includes CES-D-8 and CESD-10): Center for Epidemiologic Studies Depression Scale; BI: Barthel Index; GHQ-12 (and GHQ-12/28): General Health Questionnaire; MMSE: Mini-Mental State Examination; CDR: Clinical Dementia Rating; EQ-5D: EuroQol 5-Dimension; SF-36/SF-12: Short-Form Health Survey; ILD: Interstitial Lung Disease; WHO-5: World Health Organization Five Well-Being Index; WHOQOL-BREF: World Health Organization Quality of Life (brief version); QoL/HRQoL: Quality of Life/Health-Related Quality of Life; SHARE: Survey of Health, Ageing and Retirement in Europe; MAPLe: Method for Assigning Priority Levels; CWBI: Caregiver Wellbeing Index; interRAI: International Resident Assessment Instrument; CASP/CASP-19: Control, Autonomy, Self-Realisation, Pleasure scale; HADS: Hospital Anxiety and Depression Scale; ADL/Katz ADL: Activities of Daily Living; SWLS: Satisfaction with Life Scale; PCS: Physical Component Score/Preparedness for Caregiving Scale; IADL/Lawton IADL: Instrumental Activities of Daily Living; NSOC: National Study of Caregiving; PSS: Perceived Stress Scale; MCS: Mental Component Score; UCLA: UCLA Loneliness Scale; EURO-D: European Depression Scale; HC: Home Care; RCT: Randomized Controlled Trial; TAP: Tailored Activity Program; HHI: Herth Hope Index; RTBPPT: Reminiscence Therapy Based on Positive Psychology Theory; VR-12: Veterans RAND 12 Item Health Survey; GAD-7: Generalized Anxiety Disorder 7.

## Data Availability

Data are contained within the article.

## Supplementary Materials

The following supporting information can be downloaded at: https://www.mdpi.com/article/10.3390/ejihpe16090129/s1. Table S1: PRISMA 2020 Checklist; Supplementary Material S1: Search strategy; Table S2A: Quality of the studies included in the review assessed using the Joanna Briggs Institute Critical Appraisal Tools; Supplementary Table S2B: Mixed methods appraisal tool (MMAT); Table S3: Full-text articles excluded and reasons for exclusion.

## References

[B1-ejihpe-16-00129] Alvarado García A. M., Salazar Maya Á. M. (2014). Análisis del concepto de envejecimiento. Gerokomos.

[B2-ejihpe-16-00129] Alvira M. C., Risco E., Cabrera E., Farré M., Hallberg I. R., Bleijlevens M. H. C., Meyer G., Koskenniemi J., Soto M. E., Zabalegui A. (2015). The association between positive-negative reactions of informal caregivers of people with dementia and health outcomes in eight European countries: A cross-sectional study. Journal of Advance Nursing.

[B3-ejihpe-16-00129] Ankuda C. K., Maust D. T., Kabeto M. U., McCammon R. J., Langa K. M., Levine D. A. (2017). Association between spousal caregiver well-being and care recipient healthcare expenditures. Journal of the American Geriatrics Society.

[B4-ejihpe-16-00129] Betini R. S. D., Hirdes J. P., Curtin-Telegdi N., Gammage L., Vansickle J., Poss J. (2018). Development and validation of a screener based on interRAI assessments to measure informal caregiver wellbeing in the community. BMC Geriatrics.

[B5-ejihpe-16-00129] Bongelli R., Busilacchi G., Pacifico A., Fabiani M., Guarascio C., Sofritti F., Lamura G., Santini S. (2024). Caregiving burden, social support, and psychological well-being among family caregivers of older Italians: A cross-sectional study. Frontier Public Health.

[B6-ejihpe-16-00129] Brandt M., Kaschowitz J., Quashie N. T. (2022). Socioeconomic inequalities in the wellbeing of informal caregivers across Europe. Aging & Mental Health.

[B7-ejihpe-16-00129] Chen H., Fang Y., Ma L., Jiang W., Luo F., Cai S., Li Z., Fu L., Yin Z. (2025). The mediating role of resilience between dyadic perceived social support and depression at the older adults with disabilities and their family caregivers in China: An actor–partner interdependence model extended to mediation. Asian Nursing Research (Korean Society of Nursing Science).

[B8-ejihpe-16-00129] Chen J., Xiao H., Chen Y., Sun H., Chen S., Zheng J. (2020). Effect of reminiscence therapy based on positive psychology theory (RTBPPT) on the positive feelings of the spousal caregivers of elderly patients with advanced cancer in China. Eurean Journal of Cancer Care.

[B9-ejihpe-16-00129] Crespo M., Fernández-Lansac V. (2015). Resiliencia en cuidadores familiares de personas mayores dependientes. Anales de Psicologia.

[B10-ejihpe-16-00129] Dionne-Odom J. N., Demark-Wahnefried W., Taylor R. A., Rocque G. B., Azuero A., Acemgil A., Martin M. Y., Astin M., Ejem D., Kvale E., Heaton K., Pisu M., Partridge E. E. (2017). The self-care practices of family caregivers of persons with poor prognosis cancer: Differences by varying levels of caregiver well-being and preparedness. Supportive Care in Cancer.

[B11-ejihpe-16-00129] Fang L., Dong M., Fang W., Zheng J. (2022). Relationships between care burden, resilience, and depressive symptoms among the main family caregivers of stroke patients: A cross-sectional study. Frontier Psychiatry.

[B12-ejihpe-16-00129] Freedman V. A., Cornman J. C., Carr D., Lucas R. E. (2019). Time use and experienced wellbeing of older caregivers: A sequence analysis. Gerontologist.

[B13-ejihpe-16-00129] Gamble L. D., Parker S., Quinn C., Bennett H. Q., Martyr A., Sabatini S., Pentecost C., Collins R., Dawson E., Hunt A., Allan L., Burns A., Litherland R., Victor C., Matthews F. E., Clare L. (2022). A comparison of well-being of carers of people with dementia and their ability to manage before and during the COVID-19 pandemic: Findings from the IDEAL study. Journal of Alzheimer’s Disease.

[B14-ejihpe-16-00129] Giebel C. M., Sutcliffe C. (2018). Initiating activities of daily living contributes to well-being in people with dementia and their carers. International Journal of Geriatric Psychiatry.

[B15-ejihpe-16-00129] Gitlin L. N., Marx K., Piersol C. V., Hodgson N. A., Huang J., Roth D. L., Lyketsos C. (2021). Effects of the tailored activity program (TAP) on dementia-related symptoms, health events and caregiver wellbeing: A randomized controlled trial. BMC Geriatrics.

[B16-ejihpe-16-00129] Grosbois J. M., Gephine S., Kyheng M., Le Rouzic O., Chenivesse C. (2022). Improving the wellbeing of caregivers of patients with COPD using a home-based pulmonary rehabilitation programme. ERJ Open Research.

[B17-ejihpe-16-00129] Huenchuan S., Jaspers-Faijer D. (2011). Los derechos de las personas mayores: Materiales de estudio y divulgación. Módulo 1.

[B18-ejihpe-16-00129] Hwang Y., Kim J. (2024). Influence of caregivers’ psychological well-being on the anxiety and depression of care recipients with dementia. Geriatric Nursing.

[B19-ejihpe-16-00129] Instituto de Mayores y Servicios Sociales (IMSERSO) (2005). Cuidados a las personas mayores en los hogares Españoles: El entorno familiar.

[B20-ejihpe-16-00129] Joling K. J., Windle G., Dröes R. M., Meiland F., van Hout H. P. J., Vroomen J. M., van de Ven P. M., Moniz-Cook E., Woods B. (2016). Factors of resilience in informal caregivers of people with dementia from integrative international data analysis. Dementia and Geriatric Cognitive Disorders.

[B21-ejihpe-16-00129] Kamalpour M., Rezaei Aghdam A., Watson J., Tariq A., Buys L., Eden R., Rehan S. (2021). Online health communities, contributions to caregivers and resilience of older adults. Health and Social Care in the Community.

[B22-ejihpe-16-00129] LaManna J. B., Unruh L., Chisholm L., Pericles P., Fotovvat H. (2020). Perceptions of health and well-being among older adult caregivers: Comparisons of current caregivers with former and never caregivers. Geriatric Nursing.

[B23-ejihpe-16-00129] Lee D. C. A., Burton E., Slatyer S., Jacinto A., Oliveira D., Bryant C., Khushu A., Tiller E., Lalor A., Watson M., Layton N., Brusco N., Hill K. D. (2022). Understanding the role, quality of life and strategies used by older carers of older people to maintain their own health and well-being: A national Australian survey. Clinical Intervention in Aging.

[B24-ejihpe-16-00129] Li F., Parsons J., Cheung G. (2024). Exploring the support needs of Chinese family carers of people living with dementia in New Zealand during the COVID-19 pandemic: A resilience resources framework perspective. International Journal of Environment Research and Public Health.

[B25-ejihpe-16-00129] Litzelman K., Kent E. E., Mollica M., Rowland J. H. (2016). How does caregiver well-being relate to perceived quality of care in patients with cancer? Exploring associations and pathways. Journal of Clinical Oncology.

[B26-ejihpe-16-00129] Lu N., Liu J., Lou V. W. Q. (2015). Caring for frail elders with musculoskeletal conditions and family caregivers’ subjective well-being: The role of multidimensional caregiver burden. Archives of Gerontology Geriatrics.

[B27-ejihpe-16-00129] Martyr A., Rusted J. M., Quinn C., Gamble L. D., Collins R., Morris R. G., Clare L. (2023). Resilience in caregivers of people with mild-to-moderate dementia: Findings from the IDEAL cohort. BMC Geriatrics.

[B28-ejihpe-16-00129] Ottmann G., Maragoudaki M. (2015). Fostering resilience later in life: A narrative approach involving people facing disabling circumstances, carers and members of minority groups. Ageing & Society.

[B29-ejihpe-16-00129] Özkan Tuncay F. (2024). The relationship between preparedness for caregiving and spiritual well-being in the carers of stroke patients: A case study in Türkiye. Journal of Religion and Health.

[B30-ejihpe-16-00129] Page M. J., McKenzie J. E., Bossuyt P. M., Boutron I., Hoffmann T. C., Mulrow C. D., Shamseer L., Tetzlaff J. M., Akl E. A., Brennan S. E. (2021). The PRISMA 2020 statement: An updated guideline for reporting systematic reviews. BMJ.

[B31-ejihpe-16-00129] Quinn C., Nelis S. M., Martyr A., Victor C., Morris R. G., Clare L. (2019). Influence of positive and negative dimensions of dementia caregiving on caregiver well-being and satisfaction with life: Findings from the IDEAL study. American Journal of Geriatric Psychiatry.

[B32-ejihpe-16-00129] Quinn C., Toms G. (2019). Influence of positive aspects of dementia caregiving on caregivers’ well-being: A systematic review. The Gerontologist. Gerontological Society of America.

[B33-ejihpe-16-00129] Schneider U., Kleindienst J. (2016). Monetising the provision of informal long-term care by elderly people: Estimates for European out-of-home caregivers based on the well-being valuation method. Health and Social Care in the Community.

[B34-ejihpe-16-00129] Shah R. J., Collard H. R., Morisset J. (2018). Burden, resilience and coping in caregivers of patients with interstitial lung disease. Heart and Lung.

[B35-ejihpe-16-00129] Sheehan O. C., Haley W. E., Howard V. J., Huang J., Rhodes J. D., Roth D. L. (2021). Stress, burden, and well-being in dementia and nondementia caregivers: Insights from the caregiving transitions study. The Gerontologist.

[B36-ejihpe-16-00129] Singh Solorzano C., Leigh E., Steptoe A., Ronaldson A., Kidd T., Jahangiri M., Poole L. (2021). The impact of caregiving burden on mental well-being in coronary artery bypass graft surgery caregivers: The mediatory role of perceived social support. International Journal of Environment Research and Public Health.

[B37-ejihpe-16-00129] Smagula S. F., Beach S., Rosso A. L., Newman A. B., Schulz R. (2017). Brain structural markers and caregiving characteristics as interacting correlates of caregiving strain. American Journal of Geriatric Psychiatry.

[B38-ejihpe-16-00129] Spasova S., Baeten R., Vanhercke B. (2018). Challenges in long-term care in Europe. Eurohealth.

[B39-ejihpe-16-00129] Tkatch R., Bazarko D., Musich S., Wu L., MacLeod S., Keown K., Hawkins K., Wicker E. (2017). A pilot online mindfulness intervention to decrease caregiver burden and improve psychological well-being. Journal of Evidence-Based Integrative Medicine.

[B40-ejihpe-16-00129] Ungar M. (2011). The social ecology of resilience: Addressing contextual and cultural ambiguity of a nascent construct. American Journal of Orthopsychiatry.

[B41-ejihpe-16-00129] Van Hout E., Peters S., Jansen L., Rober P., van den Akker M. (2020). An exploration of spousal caregivers’ well-being after the death of their partners who were older cancer patients—A phenomenological approach. European Journal of Oncology Nursing.

[B42-ejihpe-16-00129] van Roij J., Brom L., Sommeijer D., van de Poll-Franse L., Raijmakers N. (2021). Self-care, resilience, and caregiver burden in relatives of patients with advanced cancer: Results from the eQuiPe study. Supportive Care in Cancer.

[B43-ejihpe-16-00129] Wagner M., Brandt M. (2018). Long-term care provision and the well-being of spousal caregivers: An analysis of 138 European regions. Journals of Gerontology—Series B Psychological Sciences and Social Sciences.

[B44-ejihpe-16-00129] White J., Falcioni D., Barker R., Bajic-Smith J., Krishnan C., Mansfield E., Hullick C. (2024). Persisting gaps in dementia carer wellbeing and education: A qualitative exploration of dementia carer experiences. Journal of Clinical Nursing.

[B45-ejihpe-16-00129] World Health Organization (2020). Decade of healthy ageing: Baseline report.

[B46-ejihpe-16-00129] Xiong Z., Wu X., Yang J., Zou J., Zhong Q., Dai Y., Zhang Q., Wang A. (2025). Association and interaction between resilience and psychological distress in disabled elderly–caregiver dyads: An actor-partner interdependence model. Geriatric Nursing.

[B47-ejihpe-16-00129] Yan E., Ng H. K. L., Sun R., Lai D. W. L., Cheng S. T., Lou V. W. Q., Fong D. Y. T., Kwok T. (2022). Resilience as a protective factor against elder abuse by family caregivers: Findings from a cross-sectional study in Hong Kong. Journal of Adult Protection.

