# Supplementary material for: Resilience and Protective Factors Associated with Well-Being Among Older Informal Caregivers: A Convergent Segregated Mixed Studies Systematic Review
Source: Eur J Investig Health Psychol Educ. 2026 Aug 28;16(9):129. doi: 10.3390/ejihpe16090129 (PMC13605886; doi:10.3390/ejihpe16090129)
Supplement: Supplementary file 1 [file ejihpe-16-00129-s001.zip › ejihpe-4442366-supplementary (Tables).pdf]

Material Supplementary S1:

PubMed (Search Date: September 10, 2025) ((caregiver*[tiab] OR "informal care"[tiab] OR carer*[tiab] OR "family caregiver"[tiab]) AND (resilience[tiab] OR resiliency[ti] OR "positive psychology"[tiab] OR wellbeing[tiab] OR "well-being"[tiab] OR "posttraumatic growth"[tiab])) AND (elderly[tiab] OR "older adult*"[tiab] OR "65 years"[tiab] OR aged[tiab]) NOT ("professional caregiver*"[ti] OR "formal caregiver*"[ti]) *Filters: Last 10 years, English, Spanish.--> 36.*

Scopus (Search Date: September 12, 2025) (TITLE-ABS-KEY(caregiver* OR "informal care" OR carer* OR "family caregiver") AND TITLE-ABS-KEY(resilience OR resilienc* OR "positive psychology" OR wellbeing OR "well-being" OR "posttraumatic growth") AND TITLE-ABS-KEY(elderly OR "older adult*" OR "65 years" OR aged) AND NOT TITLE("professional caregiver*" OR "formal caregiver*")) *Filters: Last 10 years, English, Spanish.-->7298.*

CINAHL via EBSCO (Search Date: September 11, 2025) (TI (caregiver* OR "informal care" OR carer* OR "family caregiver") OR AB (caregiver* OR "informal care" OR carer* OR "family caregiver")) AND (TI (resilience OR resiliency OR "positive psychology" OR wellbeing OR "well-being" OR "posttraumatic growth") OR AB (resilience OR resiliency OR "positive psychology" OR wellbeing OR "well-being" OR "posttraumatic growth")) AND (MW (elderly OR "older adult*" OR "65 years" OR aged)) NOT TI ("professional caregiver*" OR "formal caregiver*") *Filters: Last 10 years, English, Spanish.-->1505.*

PsycINFO via EBSCO (Search Date: September 29, 2025) (TI (caregiver* OR "informal care" OR carer* OR "family caregiver") OR AB (caregiver* OR "informal care" OR carer* OR "family caregiver")) AND (TI (resilience OR resiliency OR "positive psychology" OR wellbeing OR "well-being" OR "posttraumatic growth") OR AB (resilience OR resiliency OR "positive psychology" OR wellbeing OR "well-being" OR "posttraumatic growth")) AND (MW (elderly OR "older adult*" OR "65 years" OR aged)) NOT TI ("professional caregiver*" OR "formal caregiver*") *Filters: Last 10 years, English, Spanish ➔725.*

Supplementary Table S2A: Quality of the studies included in the review assessed using the Joanna Briggs Institute Critical Appraisal Tools

**Author, year**	**Q1**	**Q2**	**Q3**	**Q4**	**Q5**	**Q6**	**Q7**	**Q8**	**Q9**	**Q10**	**Q11**	**Q12**	**Q13**	**Score (%)**
Cohort Studies														
Ankuda CK, et al, 2017	Y	Y	Y	Y	Y	Y	Y	Y	Y	N	Y	-----	-----	10/11 (90.9 %)
Brandt M, et al, 2022	U	Y	Y	Y	Y	Y	Y	Y	U	N	Y	-----	-----	9/11 (81.81 %)
Gamble, L. D. et al., 2022	Y	Y	Y	Y	Y	N/A	Y	Y	U	N	Y	-----	-----	8.5/11 (77.27 %)
Betini et al., 2018	N	Y	Y	Y	Y	Y	Y	U	Y	Y	Y	-----	-----	9.5/11 (86.36 %)
Litzelman K, et al, 2016	U	Y	Y	Y	Y	Y	N	Y	U	Y	Y	-----	-----	9/11 (81.81 %)
Singh Solorzano; et al., 2021	Y	Y	Y	Y	Y	N	Y	Y	N	N	Y	-----	-----	8/11 (72.7%)
RCT														
Gitlin, L. N., et al., 2021	Y	Y	Y	N	N	Y	U	N	Y	Y	Y	Y	Y	9.5/13 (73.07 %)
Chen, J, et al., 2020	Y	N	Y	N	N	Y	U	Y	Y	Y	Y	Y	Y	9.5/13 (73.07 %)
Quasi-experimental Studies														
Tkatch R, et al,2017	Y	N	Y	N/A	Y	Y	Y	Y	Y	-----	-----	-----	-----	7/9 (77.78 %)
Grosbois JM, et al. 2022	Y	N	U	Y	Y	Y	Y	Y	Y	-----	-----	-----	-----	7.5/9 (83.3 %)
Cross-sectional Studies														
Lee D, et al, 2022	Y	Y	Y	Y	Y	Y	Y	Y	-----	-----	-----	-----	-----	8/8 (100 %)
Dionne-Odom, et al, 2017	Y	Y	Y	Y	Y	N	Y	Y	-----	-----	-----	-----	-----	7/8 (87.5 %)

H. Chen et al., 2025	Y	Y	Y	Y	Y	Y	Y	Y	-----	-----	-----	-----	-----	8/8 (100 %)
LaManna, et al, 2020	Y	Y	Y	Y	Y	Y	U	Y	-----	-----	-----	-----	-----	7.5/8 (93.75 %)
Nan Lu, et al, 2015	Y	Y	Y	Y	Y	Y	Y	Y	-----	-----	-----	-----	-----	8/8 (100 %)
Tuncay et al., 2024	Y	Y	Y	Y	Y	Y	Y	Y	-----	-----	-----	-----	-----	8/8 (100 %)
Martyr A, et al 2023	Y	Y	Y	Y	Y	Y	Y	Y	-----	-----	-----	-----	-----	8/8 (100 %)
Yan E, et al, 2022	Y	Y	Y	Y	Y	Y	Y	Y	-----	-----	-----	-----	-----	8/8 (100 %)
Fang L; et al 2022	Y	Y	Y	Y	Y	Y	Y	Y	-----	-----	-----	-----	-----	8/8 (100 %)
Sheehan, et al. 2021	Y	Y	Y	Y	Y	Y	Y	Y	-----	-----	-----	-----	-----	8/8 (100 %)
Freedman V, et al., 2019	Y	Y	Y	Y	Y	Y	Y	Y	-----	-----	-----	-----	-----	8/8 (100 %)
Quinn C et al, 2019	Y	Y	Y	Y	Y	Y	Y	Y	-----	-----	-----	-----	-----	8/8 (100 %)
Wagner M, et al; 2018	Y	Y	U	Y	Y	Y	Y	Y	-----	-----	-----	-----	-----	7.5/8 (93.75 %)
Giebel, et al., 2018	Y	Y	Y	Y	N	N	Y	Y	-----	-----	-----	-----	-----	6/8 (75 %)
Joling K.J., et al., 2016	Y	Y	Y	Y	Y	Y	Y	Y	-----	-----	-----	-----	-----	8/8 (100%)
Alvira M, et al, 2015	Y	Y	Y	Y	U	N	Y	Y	-----	-----	-----	-----	-----	6.5/8 (81.25 %)
Schneider, U; et al, 2016	Y	Y	U	U	Y	Y	Y	Y	-----	-----	-----	-----	-----	7/8 (87.5 %)

Qualitative Studies													
White et al., 2024	Y	Y	Y	Y	Y	Y	Y	Y	Y	Y	-----	-----	-----	10/10 (100 %)
Li F.; et al 2024	Y	Y	Y	Y	Y	Y	Y	Y	Y	Y	-----	-----	-----	10/10 (100 %)
Van Hout, et al., 2020	Y	Y	Y	Y	Y	Y	Y	Y	Y	Y	-----	-----	-----	10/10 (100 %)

Y:Yes; N: No; U: unclear; N/A: Not Applicable; RCT: Randomized Controlled Trial

Cohort Studies: Q1. Were the two groups similar and recruited from the same population?; Q2. Were the exposures measured similarly to assign people to both exposed and unexposed groups?; Q3. Was the exposure measured in a valid and reliable way?;Q4. Were confounding factors identified?;Q5. Were strategies to deal with confounding factors stated?; Q6. Were the groups/participants free of the outcome at the start of the study (or at the moment of exposure)?; Q7. Were the outcomes measured in a valid and reliable way?; Q8. Was the follow up time reported and sufficient to be long enough for outcomes to occur?;Q9. Was follow up complete, and if not, were the reasons to loss to follow up described and explored?;Q10. Were strategies to address incomplete follow up utilized?;Q11. Was appropriate statistical analysis used?

RCT: Q1. Was a genuine randomization method applied to assign participants to their respective treatment groups?; Q2. Was the allocation of participants to the study groups kept concealed?; Q3. Were the different treatment groups comparable at the baseline of the trial?; Q4. Were the participants kept unaware of (blinded to) their treatment assignment?; Q5. Were the personnel administering the interventions blinded to the treatment allocation?; Q6. Were the individuals assessing the outcomes blinded to the participants' group assignment?; Q7. Aside from the specific intervention being investigated, were all study groups treated identically?; Q8. Was participant follow-up complete, and if not, were any differences in follow-up between the groups adequately described and analyzed?; Q9. Were participants analyzed within the specific groups to which they were initially randomized (intention-to-treat analysis)?; Q10. Were the outcome measures assessed in an identical manner across all treatment groups?; Q11. Were the outcomes measured using reliable methods and tools?; Q12. Was an appropriate statistical analysis applied to the data?; Q13. Was the overall trial design suitable, and were any deviations from a standard RCT framework (such as individual randomization or parallel groups) properly accounted for during the execution and analysis of the trial?

Cross-sectional Studies: Q1. Were the inclusion criteria for the study sample clearly defined?; Q2. Were the study participants and the research setting described in sufficient detail?; Q3. Was the exposure evaluated using valid and reliable methods?; Q4. Were standard, objective criteria applied to measure the condition being studied?;Q5. Were potential confounding variables identified?;Q6. Were specific strategies outlined to address these confounding factors?;Q7. Were the outcomes assessed using valid and reliable instruments?;Q8. Was a suitable statistical analysis applied to the data?

Quasi-experimental Studies: Q1. Is it clear within the study what constitutes the 'cause' and the 'effect' (i.e., is there no ambiguity regarding which variable occurs first)?;Q2. Was a control group included in the study?;Q3. Were the participants included in the comparisons similar at baseline?;Q4. Did the participants involved in the comparisons receive similar treatment or care, aside from the specific intervention or exposure of interest?;Q5. Were multiple outcome measurements taken both before and after the intervention or exposure?;Q6. Were the outcomes assessed in the same manner for all participants included in the comparisons?;Q7. Were the outcomes measured in a reliable way?;Q8. Was participant follow-up complete, and if not, were any differences in follow-up between the groups properly described and analyzed?;Q9. Was a suitable statistical analysis applied to the data?

Qualitative Studies:Q1. Is there congruence between the stated philosophical perspective and the chosen research methodology?;Q2. Is there congruence between the research methodology and the research questions or objectives?;Q3. Is there congruence between the research methodology and the methods used for data collection?;Q4. Is there congruence between the research methodology and how the data is represented and analyzed?;Q5. Is there congruence between the research methodology and the interpretation of the results?;Q6. Is there a clear statement locating the researcher culturally or theoretically?;Q7. Is the mutual influence between the researcher and the research process adequately addressed?;Q8. Are the participants and their perspectives adequately represented?;Q9. Is the research ethical according to current standards, or for recent studies, is there evidence of ethical approval from an appropriate regulatory body?;Q10. Do the conclusions drawn in the research report clearly flow from the analysis or interpretation of the data?

Supplementary Table S2B: Mixed methods appraisal tool (MMAT)

Mixed-methods																		
Shah RJ, al., 2018	S1	S2	Q1.1	Q1.2	Q1.3	Q1.4	Q1.5	Q4.1	Q4.2	Q4.3	Q4.4	Q4.5	Q5.1	Q5.2	Q5.3	Q5.4	Q5.5	**Score (%)**
	Y	Y	Y	Y	Y	Y	Y	Y	N	Y	N	Y	Y	Y	Y	Y	Y	95.0% (MMAT: 5/5)

S1 and S2: Screening questions. S1. Are there clear research questions?; S2. Do the collected data allow to address the research questions?
Category 1: Qualitative: Q1.1. Is the qualitative approach appropriate to answer the research question?; Q1.2. Are the qualitative data collection methods adequate to address the research question?; Q1.3. Are the findings adequately derived from the data?; Q1.4. Is the interpretation of results sufficiently substantiated by data?; Q1.5. Is there coherence between qualitative data sources, collection, analysis and interpretation?.
Category 4: Quantitative Descriptive:Q4.1. Is the sampling strategy relevant to address the research question?; Q4.2. Is the sample representative of the target population?; Q4.3. Are the measurements appropriate?; Q4.4. Is the risk of nonresponse bias low?; Q4.5. Is the statistical analysis appropriate to answer the research question?
Category 5: Mixed Methods: Q5.1. Is there an adequate rationale for using a mixed methods design to address the research question?; Q5.2. Are the different components of the study effectively integrated to answer the research question?; Q5.3. Are the outputs of the integration of qualitative and quantitative components adequately interpreted?; Q5.4. Are divergences and inconsistencies between quantitative and qualitative results adequately addressed?; Q5.5. Do the different components of the study adhere to the quality criteria of each tradition?

Supplementary Table S3: Full-text articles excluded and reasons for exclusion

Author & Year	Title of the Study	Methodological Reason for Exclusion
**Bongelli R, et al., 2024**	Caregiving burden, social support, and psychological well-being among family caregivers of older Italians: a cross-sectional study	Ineligible Phenomenon of Interest: Focuses broadly on general caregiving burden and coping without evaluating the specific late-life resilience process or assets.
**Hwang Y, & Kim J., 2024**	Influence of caregivers' psychological well-being on the anxiety and depression of care recipients with dementia.	Ineligible Outcome: Focuses primarily on the dyadic impact of caregiver well-being on the recipient's anxiety/depression rather than the caregiver's own resilience.
**Lappalainen P, et al., 2019**	CareACT – internet-based intervention for enhancing the psychological well-being of elderly caregivers – a study protocol of a controlled trial	Ineligible Study Design: Published protocol for a randomized controlled trial; lacks empirical outcome data or baseline/post-intervention scores.
**Xiong Z, et al., 2025**	Association and interaction between resilience and psychological distress in disabled elderly–caregiver dyads: An actor-partner interdependence model.	Ineligible Population (Age Constraint): Caregiver sample mean age is below the eligibility threshold (55.73 ± 15.68 years) and findings are not disaggregated for the ≥65 subgroup.
**Rababa M, et al., 2023**	Environmental and Caregivers-Related Factors Influencing the Psychosocial Well-Being of Older Adults with Dementia: A Systematic Review.	Ineligible Study Design: Secondary literature (systematic review). Excluded to prevent data duplication and double-counting of primary studies.
**Kamalpour M,et al., 2021**	Online health communities, contributions to caregivers and resilience of older adults.	Ineligible Study Design: Secondary literature (systematic review). Excluded to prevent data duplication and double-counting of primary studies.
**Zeyu Liu, et al., 2024**	Care stressors and perceived stress among family caregivers of Chinese older adults with disabilities: The mediation effect of resilience	Ineligible Population: Target sample consists of caregivers with a mean age under 65 years with no isolated data for late-life caregiving.
**Ferraris G, et al., 2022**	Dyadic associations between perceived social support and psychological well-being in caregivers and older care recipients	Ineligible Population (Age Constraint): Caregiver mean age is under 65 (mean = 62.8 years) with no age-disaggregated analysis to isolate findings for older caregivers.
**Ottmann G, & Maragoudaki M., 2015**	Fostering resilience later in life: a narrative approach involving people facing disabling circumstances, carers and members of minority groups.	Ineligible Population (Missing Demographic Data): Qualitative narrative study targeting individuals over 60, but lacks specific confirmation of the caregivers' mean age or age-specific findings for those ≥65.
**Enya Redican, et al., 2025**	The prevalence, characteristics, and psychological wellbeing of unpaid carers in the United Kingdom	Ineligible Population (Age Constraint): Large mixed-age survey; fails to report disaggregated or subgroup findings specifically for caregivers aged 65 and older.

**McGarrigle CA, et al., 2022**	Caring in the time of COVID-19, longitudinal trends in well-being and mental health in carers in Ireland: Evidence from the Irish Longitudinal Study on Ageing (TILDA).	Ineligible Outcome: Focuses on general longitudinal trends in stress and depression during COVID-19 without direct measurement of the resilience process or protective assets.
**Liu Y, & Hughes MC., 2024**	Financial strain, health behaviors, and psychological well-being of family caregivers of older adults during COVID-19	Ineligible Population (Age Constraint): Caregiver sample mean age is below eligibility criteria (mean = 59 years) with no age-disaggregated data.
**Maggie Man-Sin Lee, et al., 2024**	Caregiver Employees' Mental Well-Being in Hong Kong	Ineligible Population (Employment Focus): Focuses on working caregiver employees of mixed ages and fails to isolate or report demographics and scores for caregivers aged 65 and older.
**Wang H, et al., 2025**	Relationship between sense of coherence and subjective well-being among family caregivers of breast cancer patients: a latent profile analysis	Ineligible Population: Caregivers are primarily younger to middle-aged adults with no isolated data for the older subgroup.
**Atzeni M, et al., 2024**	Reliability and Factor Structure of the Well-Being and Respect for Human Rights Questionnaire in Measuring Caregivers' Perception	Ineligible Population (Age Constraint): Caregiver mean age is 55 (SD = 13) with no distinct findings or disaggregation for older adult caregivers.
**van Roij J, et al., 2021**	Self-care, resilience, and caregiver burden in relatives of patients with advanced cancer: results from the eQuiPe study	Ineligible Population (Age Constraint): Caregiver mean age is 61 years (range 18–87); findings do not present separate analyses for caregivers over 65 years.
**Cathryn Pinto, et al., 2021**	Emotional distress and well-being among people with motor neurone disease (MND) and their family caregivers: a qualitative interview study.	Methodological Deficit: The primary standardized outcome questionnaire (ALSAQ-5) was exclusively administered to the care recipients, leaving caregiver outcomes unmeasured.
**Ahrang Jung, et al., 2021**	Well-being and Perceptions of Supportive Resources among Caregivers of Patients with Bladder Cancer	Ineligible Population (Age Constraint): Caregiver sample mean age is below eligibility criteria (mean = 61.4 years) with no subgroup analysis for older caregivers.
**Lee Y, et al., 2020**	Psychological Well-Being Among Informal Caregivers in the Canadian Longitudinal Study on Aging: Why the Location of Care Matters	Ineligible Population (Age Constraint): Caregiver mean age is under 65 (mean = 59.1 years) without separate data extraction for the older cohort.
**Karlsson T, et al., 2020**	Well-Being of Caregivers of Patients with Laryngeal Cancer Treated by Radiotherapy	Ineligible Population (Age Constraint): Caregiver sample mean age is 56.3 ± 13.1 years, failing the age criteria.
**Patricia Dias Francisquini, et al., 2020**	Relationship between well-being, quality of life and hope in family caregivers of schizophrenic people	Ineligible Population (Age Constraint): Caregiver sample mean age is under 65 (mean = 56.7 ± 15.7 years) with no age-disaggregated findings.

**Tyler CM, et al., 2020**	Structural Equation Modeling of Parkinson's Caregiver Social Support, Resilience, and Mental Health: A Strength-Based Perspective	Ineligible Population (Age Constraint): Caregiver sample mean age is 59.92 ± 14.68 years and fails to provide age-specific outcomes.
**Hou WK, et al., 2018**	Do concordances of social support and relationship quality predict psychological distress and well-being of cancer patients and caregivers?	Ineligible Population (Age Constraint): Caregiver mean age is 52.64 ± 13.41 years, with no age-disaggregated findings.
**Roberts E, & Struckmeyer KM., 2018**	The impact of respite programming on caregiver resilience in dementia care: A qualitative examination of family caregiver perspectives	Ineligible Population (Age Constraint): Qualitative study targeting family caregivers with a mean age of 61 years and does not isolate participants aged 65 and older.
**Sterba KR, et al., 2017**	Physical and emotional well-being and support in newly diagnosed head and neck cancer patient-caregiver dyads	Ineligible Population (Age Constraint): Caregiver mean age is below threshold (mean = 58.1 years) with no disaggregated findings.
**Leach MJ, et al., 2015**	Transcendental Meditation for the improvement of health and wellbeing in community-dwelling dementia caregivers [TRANSCENDENT]: a randomised wait-list controlled trial	Methodological Deficit (Underpowered Sample): Pilot study with a very small sample size (N = 17) that does not report distinct, generalizable findings specifically for caregivers over 65.
